# Silicon Nanowires for Gas Sensing: A Review

**DOI:** 10.3390/nano10112215

**Published:** 2020-11-06

**Authors:** Mehdi Akbari-Saatlu, Marcin Procek, Claes Mattsson, Göran Thungström, Hans-Erik Nilsson, Wenjuan Xiong, Buqing Xu, You Li, Henry H. Radamson

**Affiliations:** 1 Department of Electronics Design, Mid Sweden University, Holmgatan 10, SE-85170 Sundsvall, Sweden; Claes.Mattsson@miun.se (C.M.); Goran.Thungstrom@miun.se (G.T.); Hans-Erik.Nilsson@miun.se (H.-E.N.); 2 Department of Optoelectronics, Silesian University of Technology, 2 Krzywoustego St., 44-100 Gliwice, Poland; 3 Guangdong Greater Bay Area Institute of Integrated Circuit and System, Guangzhou 510535, China; xiongwenjuan@ime.ac.cn (W.X.); xubuqing@ime.ac.cn (B.X.); liyou2019@ime.ac.cn (Y.L.); 4 Key Laboratory of Microelectronic Devices & Integrated Technology, Institute of Microelectronics, Chinese Academy of Sciences, Beijing 100029, China; 5 College of Microelectronics, University of Chinese Academy of Sciences, Beijing 100049, China

**Keywords:** silicon nanowire, gas sensor, functionalization, top-down fabrication, bottom-up fabrication, heterostructures, metal oxides

## Abstract

The unique electronic properties of semiconductor nanowires, in particular silicon nanowires (SiNWs), are attractive for the label-free, real-time, and sensitive detection of various gases. Therefore, over the past two decades, extensive efforts have been made to study the gas sensing function of NWs. This review article presents the recent developments related to the applications of SiNWs for gas sensing. The content begins with the two basic synthesis approaches (top-down and bottom-up) whereby the advantages and disadvantages of each approach have been discussed. Afterwards, the basic sensing mechanism of SiNWs for both resistor and field effect transistor designs have been briefly described whereby the sensitivity and selectivity to gases after different functionalization methods have been further presented. In the final words, the challenges and future opportunities of SiNWs for gas sensing have been discussed.

## 1. Introduction

Gas sensors play an important role in our daily life for detecting various gases which have a negative effect on the environment and human safety [1,2]. The applications of such sensors include gas pollutants, evaluation of food safety, medical approaches for recognizing illness at the initial state, human safety (flammable and explosive gases for mines and indoor applications), and the automotive and chemical industries [3,4,5,6,7]. In this field, developing high performance sensors which provide reliable data with high sensitivity is the key goal for many recent research studies [8,9]. As an example, in many developing countries, evaluating the air quality is one of the most important tasks to bring new environmental solutions to avoid severe health hazards [10,11]. The market of gas sensors is still growing worldwide and only in the USA is it expected to grow from about 1 billion USD in 2019 to 1.4 billion USD in 2024 [12]. The gas sensor market is divided mostly into electrochemical, semiconductor, solid-state/metal-oxide, infrared, catalytic, photoionization, laser, and other kinds of sensors [13]. One of the highest shares in the market is held by the solid-state/metal-oxide semiconductors segment. The most popular materials used in the gas sensors are metal oxides like SnO_2_, ZnO, WO_3_, and others. However, many different sensing materials, like conductive polymers, carbon-based materials, and material hybrids, are considered in the literature to be competitive with metal oxides in the future [14].

Devices with active material in the nano-scale have proved to be promising candidates for gas sensing applications due to their high surface to volume ratio and their comparable small physical dimension to charge screening length [15,16,17,18]. A good gas sensor is the one that shows high sensitivity and selectivity towards a specific gas. In addition, the sensor should have long-term stability and repeatability, as well as a low operating temperature, and as a result low power consumption. Moreover, providing a cost-effective fabrication process for industrial perspectives should be considered. Various nanostructures, namely nanoparticles (NPs) [19,20], nanotubes (NTs) [21,22], nanowires (NWs) [23,24], and nanosheets (NSHs) [25,26], show good sensitivity to the different gases. Among all these nanostructures, silicon nanowires (SiNWs) have demonstrated substantial advantages due to their need for relatively standard processing techniques, which allows for integration with standard complementary metal oxide semiconductor (CMOS) processes for very large scale production [27,28]. In addition, Si-based NWs’ gas sensing is more flexible for doping concentration and their surface can be chemically functionalized for the selective detection of molecules in gas phase [29].

Metal oxide nanowire (MONW) gas sensors have been recently reported as a low cost and highly sensitive material with a fast response/recovery time and simple electronic interface [30,31,32,33]. MONW gas sensors are able to detect low levels of hazardous gases like NO_x_, CO_x_, NH_3_, CH_4_, H_2_S, and SO_2_ [9,34,35]. However, the main problem with these MONWs is that they operate at high temperatures (>200 °C) or under UV irradiation due to their large bandgap [33,36]. This elevated temperature, apart from energy consumption, is the main obstacle for the devices to being integrated into circuits working at room temperature (RT) [37,38]. In MONWs, the thickness of depletion layer on the metal oxide face dramatic changes upon exposure to reducing/oxidizing gas or volatile organic compounds [39,40]. In this case, SiNWs with low bandgap (1.12 eV) have higher sensitivity and present the advantage of operating at RT [29].

The pioneer work in the field of SiNW gas sensors is published by Lieber et al. who have reported the amine- and oxide-functionalized SiNW sensors. These nanowires exhibited pH-dependent conductance with linear response over a large dynamic range [41,42]. Because of this initial work, SiNWs with different dimensions attracted more attention as gas sensors and different synthesis methods were proposed to improve the device performance and sensitivity towards a certain gas [43,44].

This paper presents an overview of recent investigations concerning the functionalization, synthesis, and applications of SiNWs for gas sensing. In the first section, we explain briefly the two main process for the fabrication of SiNWs (top-down and bottom-up). Then, more attention is given to the basic gas sensing mechanism, working principle (resistor and field effect transistors), as well as different ways for functionalization (and its influence on sensing properties and mechanisms) of SiNWs. The latest review article in the field is from 2014 [43] and, since then, a large number of interesting research works related to SiNWs gas sensors have been conducted and published. Therefore, this review article presents all important research works falling into this category with recent functionalization methods. The innovation of this work stems from the tactical choice of articles with high impact in the gas sensor field. The critical insight of this survey creates a unique knowledge and provides a deep understanding of the SiNW technology for gas sensing for the readers.

## 2. Fabrication of SiNWs

Up to now, the fabrication process of NWs is divided into two main approaches [45], i.e., bottom-up and top-down. Bottom-up techniques are realized mostly by using vapor–liquid–solid (VLS) which is generally used in semiconductor research. This method may offer high quality NWs with small sizes, down to 10 nm [46]. However, there are still some issues associated with this approach, such as random orientation, metal contamination, and the inability of integration into COMS technology, which could lead to poor device uniformity and a low fabrication yield. At the same time, top-down fabrication technique is CMOS compatible and may produce SiNWs in configurations of resistors or field effect transistors (FETs) with high precision in dimensions and possibility for scaling down to 3 nm and beyond [47,48,49,50]. The top-down approach could provide a superior ability for alignment in the nanometer scale. To produce NW arrays, top-down technologies use nanofabrication including lithography, etching, cleaning, passivation, functionalization, and metallization. The lithography technique is presented by photolithography [51,52,53], E-beam lithography [47,48,54,55], deep UV (DUV) nanoimprint lithography [53,56], and side-wall transfer lithography (STL) process [57,58]. In the following sections a survey of lithography, etching techniques, and also an investigation of contact resistance for the formation of NWs are presented.

### 2.1. Top-Down Fabrication Methods

#### 2.1.1. Lithography

##### Photolithography

Lithography has always been the most important process during integrated circuit manufacture. In general, shorter wavelength light sources are normally the trend to achieve higher image resolution. Equation (1) shows the relationship between critical dimension and light source wavelength.

(1) $$CD=\frac{k\lambda }{\mathrm{NA}}.$$

**Figure 1:**
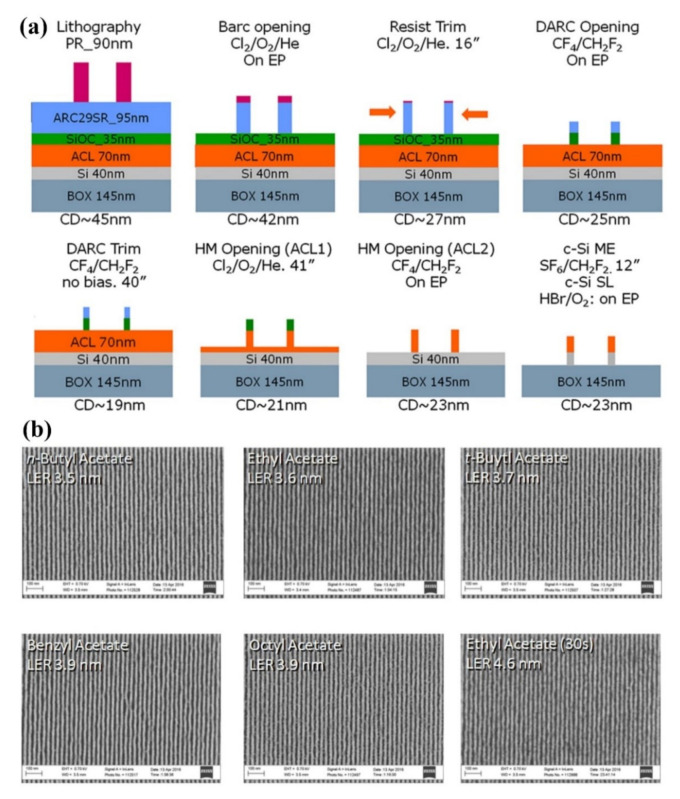
(**a**) Schematic representation of a cross-section of a 10 nm SiNW produced from SOI using a 193 nm immersion lithography process incorporating resist trimming steps and over-etching. Where HM stands for hardmask, ACL for Amorphous Carbon Layer, DRAC for Dielectric Anti-Reflective Coating, and SiOC for Silicon OxyCarbide. (**b**) CDSEM images show LER trend with increasing dose and resist quencher concentration. Figure 1b is reproduced from [68], with permission from SPIE and the author (Alex Robinson), 2017.

The critical dimension (CD) is defined as the minimum feature size that an exposure system can resolve. Here, *k* is a process dependent adjustment factor for a particular application, its range normally goes from 0.6 to about 0.8. NA presents the numerical aperture of the exposure system. It is apparent that light wavelength is critical to optimize the resolution for the NWs’ dimensions. Until now, the lithography tools have experienced five generations of development and features’ dimensions could be shrunk from the micrometer range to nanometer. The first model of lithography tool was equipped with Hg g-line emission at a wavelength of 436 nm and it could create 5–6 µm feature sizes. Later, i-line wavelength tools with 365 nm were introduced and the state of the art could approach 1 µm feature sizes. The corresponding equipment included both contact and proximity lithography, resulting in a short lifetime for the mask. A few years later, a light source of 248 nm KrF with proximity mode emerged and NWs of 0.5 µm dimension could be fabricated. This technique was further developed by new methods of the exposure system, photoresist processes, and phase shift photomasks. As a result, a feature size of 0.18~0.35 µm could be enabled. Not so long, 193-nm lithography, using an ArF light source, was introduced as commercial production, and as a result, a feature size of 65 nm and 45 nm could be successfully fabricated. At that time, many technical difficulties were solved to print 32-nm half-pitch feature by introducing immersion lithography and new photoresist materials with bottom antireflection coatings (BARCs). An innovative method was introduced through combining self-aligned double patterning (SADP) and self-aligned quadruple patterning (SAQP) techniques for fabricating 10 nm/7 nm technology modes [59]. Figure 1a demonstrates 193-nm lithography, which is still the most widely used and representative generation of lithography machine. From 436 nm g-line to 193 nm, all their light source was belonging to deep ultraviolet light [60].

In order to resolve lines (half-pitch) smaller than 7 nm, the technology had to push the exposure wavelength to the limit which is extreme ultraviolet light with a wavelength of 13.5 nm [52]. With the help of Extreme Ultraviolet (EUV) lithography system, semiconductor technology can be impelled further to beyond 3 nm and Moore’s law can be extended to more decades in the future. EUV lithography (EUVL) enables to use only a single mask exposure instead of double or quadruple exposure. There are still several issues to deal with this technique, e.g., power source, resists, and mask infrastructure [61,62]. In general, a 200-W power source is needed for processing 125 wafers per hour with size of 300-mm. Meanwhile, today, only >80 W light sources are available. Though this is not enough for large scale manufacture, the source issue is considerably mature.

For EUV photoresist sensitivity to the 13.54 nm, wavelength radiation needs to be improved, while the line-width roughness (LWR) specification has to be controlled within low several nanometers [63,64,65,66,67]. Figure 1b shows NWs made by EUVL, the LER (line edge roughness) trend with increasing dose and resist quencher concentration. The critical dimension can be well controlled around 15 nm [68].

##### E-Beam Lithography

Several lithography processes have been explored to extend UV lithography for semiconductor device manufacturing. Those are electron beam lithography (EBL), nanoimprint lithography (NIL), and Ion beam lithography (IBL). EBL is at the heart of many of these techniques and the main principle is to allow high-speed electrons to hit the surface of the photoresist to change its chemical properties. The EBL is one of the next generation photolithography technologies which attracts more attention because of its high resolution, stable performance, and relatively low costs. Instead of optical exposure, electron scanning can avoid diffraction. During exposure, an expensive mask and optical projection system are necessary, but the technique is only proper for small scale production. Because of the short wavelength of the electron, the resolution of electron beam lithography can be up to 10 nm for NW fabrication. The photoresist plays an important role in electron beam lithography technology. Currently, the commonly used electron beam photoresist includes polymethyl methacrylate (PMMA), ZEP520A and HSQ [69].

Trivedi et al. [54] demonstrated SiNW FETs fabrication using EBL. The NWs were long, but had a width less than 5 nm and exhibited high performance without employing doped junctions or high channel doping. These NW FETs showed high peak hole mobility (as high as over 1200 cm^2^/Vs), current density, and drive current, as well as a low drain leakage current and high on/off ratio.

##### Side Wall Transfer Lithography (STL)

**Figure 2:**
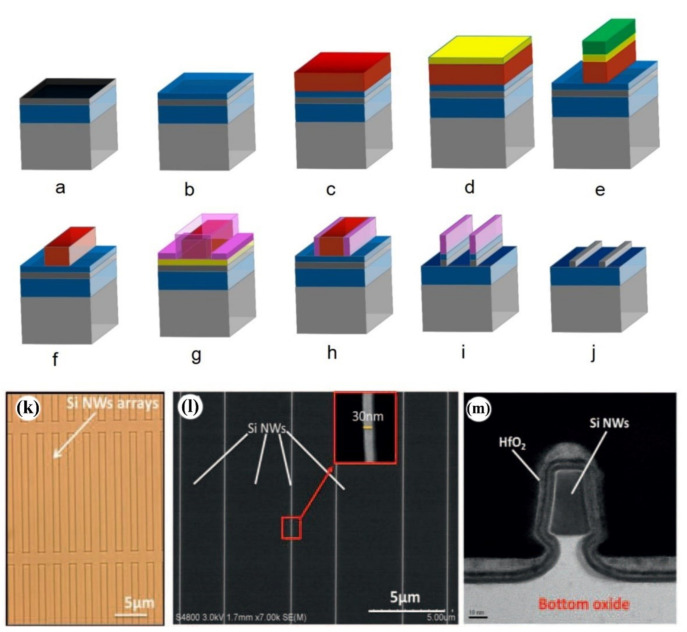
Process flow of Side-wall Transfer Lithography (STL). (**a**) SOI substrate (**b**) Oxide deposition (PEOX) on SOI, (**c**) amorphous-Si (α-Si) on PEOX, (**d**) SiN hardmask, (**e**) lithography & etch of hardmask and dummy gate, (**f**) stripe photoresist and SiN, (**g**) SiN deposit on etched α-Si, (**h**) etch spacer, (**i**) etch Si-NW, (**j**) remove α-Si and PEOX then Si-NWs are formed. Fabrication of Si NWs sensor: (**k**) top view of Si NW arrays by optical microscope, the length of NWs is about 50 µm. (**l**) SEM image of Si NW arrays, the NWs width is about 30 nm, (**m**) cross sectional TEM image of Si NW sensors, conformal and uniform HfO_2_ layer are observed, which is attributed to a good isolation between electrode and the solution of cells. Figure 2k–m are reproduced from [72], with permission from IEEE, 2020.

Side-wall transfer lithography (STL) is a kind of nanometer patterning on Si wafers scale with a resolution comparable to the best electron beam lithography [57,58]. Its advantages are CMOS compatibility, simplicity, and the realization of high density, which can be executed only without immersion, EUV, or EBL lithography. This technology only uses i-line stepper lithography to define NWs. This technique is based on the conformal deposition of silicon nitride film by low pressure chemical vapor deposition (LPCVD) over a previously patterned step in dummy gate α-Si [57,70,71] as shown in Figure 2a–j. With this technique, a minimum 10 nm width NW could be generated depending on the width of the spacer, which is determined by the thickness of deposited silicon nitride. Figure 2k–m show the SiNWs which can be used for bio- or gas-sensing applications [72].

#### 2.1.2. Etching Methods

The conventional methods to fabricate nanostructures on an Si substrate were performed by anodic (electrochemical) or stain etching in hydrofluoric acid (HF)-based solutions [73,74]. The initial method (anodization) during the last decade was replaced with metal-assisted chemical etching (MACE) owing to its simplicity and better performance. In MACE, SiNWs are fabricated by non-uniform etching of Si substrates in aqueous acid solutions, which is catalyzed by electroless deposition of metal NPs on the substrate surfaces. The nucleation of metal NPs and anisotropic etching in a solution containing HF and oxidant agent are the two main steps in this process. In order to form SiNWs on the Si substrate by MACE, two different approaches have been considered. In the first one, metal catalyst nucleation and Si etching occurs in a single solution containing of HF and metal salts (AgNO_3_, KAuCl_4_), while the second one consists of a two-step reaction involving the predeposition of metal in an aqueous solution (like HF/AgNO_3_), followed by chemical etching in the presence of HF and oxidizing agents, such as hydrogen peroxide (H_2_O_2_), nitric acid (HNO_3_), and sodium persulphate (Na_2_S_2_O_8_) [75]. Several factors affect the morphology of the grown SiNWs such as etching solution and temperature, orientation of the Si substrate, size and type of noble metal NPs, distribution of the NPs, etc. Reproducibility is the main drawback for this method. However, easy fabrication process and compatibility to create heterostructures with organic and inorganic materials are the main advantages of this method. The provided SiNWs often have a rough surface due to the lateral (side wall) etching which could affect the sensing properties of the device (later, this effect will be investigated in detail as one of the functionalization methods to increase the sensors sensitivity) [75]. In some reports, in order to achieve a predetermined size of the SiNWs, researchers use a template assisted technique (by anodized aluminum oxide) to deposit metal NPs prior to MACE [76,77].

Reactive ion etching (RIE) is another method widely used for large scale fabrication and high performance SiNW based devices. It is known as anisotropic process during which halogen radicals are utilized for dry etching of Si and SiO_2_ to form vertical array of SiNWs. In order to prevent side wall (lateral) etching, fluorine radicals from the plasma reach the Si surface to form volatile SiFx. A comprehensive study was performed by Jansen et al. [78] for the growth of SiNWs by anisotropic RIE with a mixture of SF_6_ and O_2_. This type of etching provides more precise etching compared to the wet etching. However, this technique needs to be done under vacuum to create plasma [17].

#### 2.1.3. Contact Resistance of SiNW

Compared to other low-dimensional semiconductor materials, SiNWs are widely used as different types of sensors. Meanwhile, to have a reduced contact resistance is an important issue for the electrical performance of SiNWs where any contact problem may shadow the measurements and a reasonable signal could not be obtained. In general, contact resistance of NWs (R*_contact_*) is appeared due to the resistance at the interface between the metal electrodes and SiNWs. The formation of reliable contacts, with high thermal stability, good quality Si crystalline with low resistance are the pivotal issues for nanoscale devices. By forming silicides, the contact resistance is reduced meanwhile the integration of such process is not straightforward. There are a few requirements e.g., low formation temperature, low Si consumption and high thermal stability which have to be fulfilled. For example, the thermal stability of NiSi can be tailored, when carbon is incorporated in contact windows either by epitaxy or implantation [79].

A novel approach to act with R*_contact_* is suppressing the surface Fermi-level pinning and the Schottky barrier height by tailoring the dopant profile or the interface states between the contacts and semiconductor [80,81].

To practice the idea to lower the Schottky barrier height, a considerable effort has been devoted to reduce R_*contact*_ [82] and to implement a universal and accurate model to estimate the contact resistance for a given set of contact and semiconductor resistivity values. With the requirements placed on the reduction of R_*contact*_ and dimension shrinkage of nano materials and devices, metal silicides, e.g., MnSi, CoSi_2_, PtSi, and NiSi [83], have been regarded as the standard approach for contact issues as summarized in Table 1. Among these silicides, NiSi is one of the most suitable approaches which appear to show the lowest resistance [84]. Single-crystal NiSi NW has been prepared with satisfying maximum transport current (>10^8^ A cm^−2^) and without deterioration in electric conductivity [85]. NiSi has the particular advantages compared to the other silicides listed as: appropriate work function, low thermal budget, and low consumption of Si with a more controllable process of silicide formation [86].

Sticking points to determine R*_contact_* rely on the uncertainties of the contact electrodes quality. For SiNWs formed by the bottom-up approach, the two contacts to the electrodes do not demonstrate identical performance and usually a “better” contact is formed to the root of the nanowire if compared with the tip [93]. The tip of a nanowire is expected to occupy dominant weight in total resistance since it makes contact to the Si electrode through the pinholes of the residual native oxide [94]. A common model to extract R*_contact_* is called transmission line model (TLM) [95], where the R*_contact_* is varied when the contacts are located in different distances. A depth-depletion model which takes into account the practical depletion layer under the contacts with finite depth is introduced by Smith et al. [96]. Chaudhry et al. described a technique for a fast and robust examination of the nanowire contact resistance from the in-circuit current-voltage measurements [97]. The outcome from this study shows that R*_contact_* is closely dependent on the effective conducting cross-section area where the presence of a surface depletion layer has a great impact on it. Singh et al. [98] proposed a model based on the phonon Boltzmann transport equation (BTE) in the solid and Fourier conduction to study the contact resistance of SiNWs. Their simulation operates under the assumption that Brillouin zone is isotropic, and it does not account for the dispersion, polarization, or phonon confinement effects. It is illustrated that this approach provides a good estimation of the relative magnitudes of constructed resistances, air thermal resistances, and the bulk resistance of the SiNWs on transverse heat transport.

Strong effort has been made to decrease the formation temperature of silicides. As an example, microwave annealing (MWA) has been proposed as an alternative technique to the commonly used rapid thermal annealing (RTA) [99]. The initial results showed that MWA provides at least 100 °C lower process temperatures compared to RTA. However, MWA is an impressive technique but the residual crystal after silicide formation contains a large number of defects.

Another available method for silicidation is millisecond laser annealing [100]. This technique has demonstrated excellent silicides results but the main challenge with all illumination-based annealing techniques is the surface emissivity of the substrate which has a large influence on the photon absorption. Therefore, RTA remains a popular technique for the formation of NiSi contacts.

### 2.2. Bottom-Up Fabrication Methods

One of the oldest methods for the fabrication of SiNWs is the bottom-up approach in which the Si atoms are gathered in a sequence to form SiNWs. The most commonly used bottom-up fabrication techniques for SiNW fabrication are thermal evaporation, molecular beam epitaxy (MBE), chemical vapor deposition (CVD) via a vapor-liquid-solid (VLS) process, and pulse laser deposition (PLD) [17,101].

**Figure 3:**
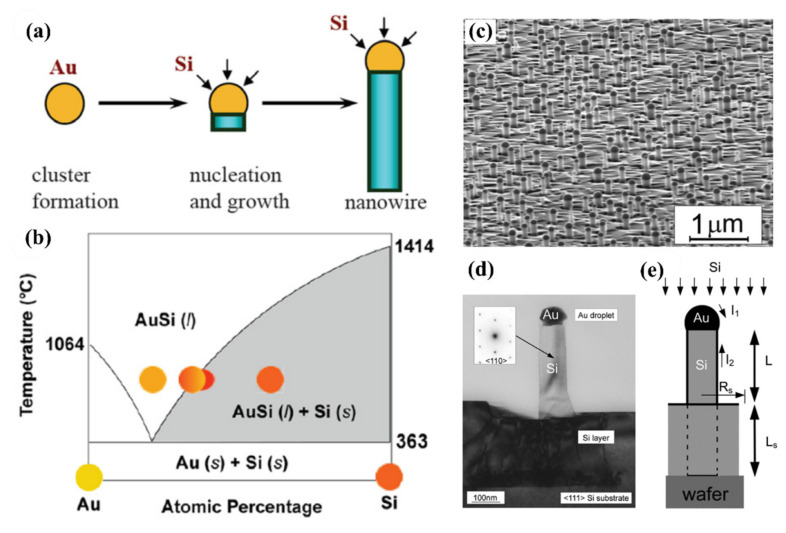
Schematic of CVD-VLS growth of SiNWs. (**a**) A liquid alloy droplet Au-Si is first formed above the eutectic temperature (363 °C) of Au and Si. The continuous feeding of Si in the vapor phase into the droplet causes supersaturation of the liquid alloy, resulting in nucleation and growth of SiNWs. (**b**) Binary phase diagram for Au and Si showing the thermodynamics of CVD-VLS growth. Reproduced from [101], with permission from IOP Publishing, 2020. (**c**) SEM images of SiNWs grown on a ⟨111⟩ Si substrate at 525 °C for 120 min by MBE. (**d**) TEM cross section image of a SiNW with Au on top. (**e**) Schematic representation of the MBE NW growth. I1 and I2 are fluxes of Si adatoms directed to the gold cap. Reproduced from [102], with permission from American Vacuum Society, 2020.

So far, the CVD has been the most popular method in bottom-up approach [101,102]. In this process, the growth of SiNWs requires a suitable noble metal (Au, Al, Cu, Fe, etc.), which serves as a catalyst. The metal nanoclusters need to be heated above the eutectic temperature for the metal-Si system in the presence of a vapor-phase source of the Si (mainly SiH_4_), resulting in a liquid droplet of the metal/Si alloy. The continuous feeding of the Si reactant into the liquid droplet supersaturates the eutectic and forms a concentration gradient between the droplet surface and the droplet/nanowire interface. Then the silicon atoms diffuse to the interface to nucleate the SiNWs (Figure 3a,b). SiH_4_, disilane (Si_2_H_6_), Si_3_H_8_**,** SiCl_4_, and dichlorosilane (SiH_2_Cl_2_) are the most frequent Si precursors in CVD growth for SiNWs. High temperatures (>800 °C) are required for decomposition of Si from precursor in chlorinated silane while for SiH_4_ is at remarkable lower temperatures [103,104]. The main drawback in this method is the metal contamination originated from catalysts which may eventually deteriorate the device performance. However, the CVD-grown SiNWs are suitable for CMOS applications due to their process compatibility.

MBE is an advanced method for fabrication of high quality SiNWs. In MBE, to supply the constituents, localized beams of particles in terms of atoms or molecules are utilized in an ultrahigh vacuum environment (below 10-10 Torr) [102]. Figure 3c presents the SEM images of SiNWs grown by MBE used Au as catalyst. This method is very similar to CVD process. In MBE, there is a Si layer deposited onto the substrate (Figure 3d) which is not used in CVD process. The MBE growth process is schematically illustrated in Figure 3e. The main drawbacks in this process, compared to the other approaches, are its slow rate, requirement of ultrahigh vacuum and the presence of an Si layer on the substrate which rarely results in the use of MBE for SiNWs growth [105].

**Figure 4:**
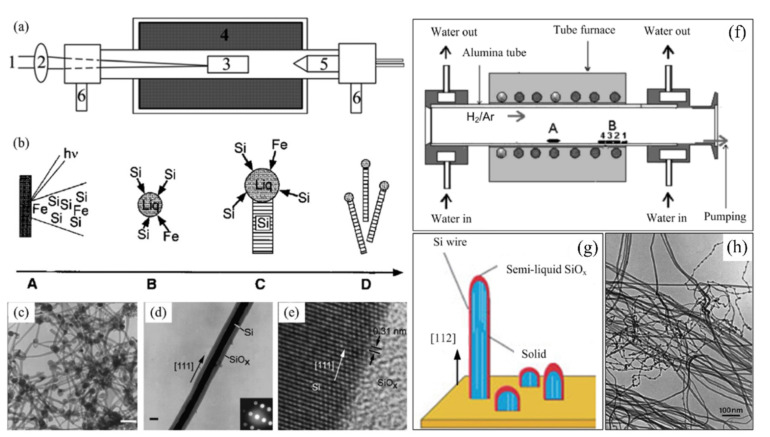
(**a**) Schematic diagram of the SiNW growth system. The output from a pulsed laser (1) is focused (2) onto a target (3) located within a quartz tube; the reaction temperature is controlled by a tube furnace (4). A cold finger (5) is utilized to collect the droplets because of the introduced carrier gas (6, left) through a flow controller and exits (6, right) into a pumping system. (**b**) Proposed PLD growth model. (**c**) TEM image of the SiNWs obtained from the cold finger. Scale bar, 100 nm. (**d**) TEM image of a SiNW; scale bar is 10 nm. (**e**) High resolution TEM image of the crystalline SiNW and amorphous SiO_x_ sheath. (**f**) Schematic diagram of the thermal evaporation system, where the SiO powder is located at A, and the grown SiNWs are located at B. (**g**) The schematic diagram of oxide-assisted growth mechanism. (**h**) TEM image showing the morphologies of randomly oriented SiNWs. Reproduced from [102], with permission from American Vacuum Society, 2020.

Another method that provides us with a well-controlled fabrication of SiNWs is laser ablation (Figure 4a). Usually, laser ablation refers to the process of removing material from a solid surface (known as target) by irradiating it with pulsed laser beam. However, if the laser intensity is high enough, it is also possible to ablate nanoparticle materials from the surface of target with a continuous wave laser [106]. For example, in the first attempt to grow SiNWs, Lieber et al. used a target made of 90% Si and 10% Fe [106]. Due to the laser irradiation, a hot vapor of Fe and Si is generated. When colliding with the inert gas molecules, the vapor species condense into small Fe-Si nanoclusters. If the temperature inside the furnace is high enough, then the Fe-Si eutectic forms. When the Fe-Si droplets get supersaturated with Si, SiNWs begin to grow and continue to grow until the nanoclusters stay in liquid and the Si reactant is sufficient. The SiNW stops to grow when the NW is not in the hot reaction zone, and the nanocluster is not in the liquid form anymore. Figure 4b shows the growth sequence (From A to D) of SiNWs. Figure 4c–e shows TEM images of SiNWs obtained from laser ablation method. The problem of pulsed laser deposition (PLD) is the high cost of operation due to the need for focused pulsed laser and high energy [107].

One of the relatively simple fabrication methods is thermal evaporation for ultra-long and large-scale production of SiNWs and it is known as oxide-assisted growth [108,109]. In this technique Si-containing powders, e.g., SiO_2_, Si, or SiO, should be evaporated at high temperatures and then carried onto the substrate. Figure 4f presents the schematic of thermal evaporation process. Due to the high temperature Si-containing powder is decomposed and the SiNWs grow. This reaction should take place inside the alumina tube furnace with an Ar/H_2_ gas mixture or a quartz tube furnace using argon [108,109]. However, this method suffers from lack of control over the orientation of NWs (Figure 4g,h) and usually ends up with a thick SiO_2_ layer on the SiNWs.

## 3. SiNWs Gas Sensing Mechanism

For sensing of a gas molecule, there are two aspects of electron transfer to be considered: reducing and oxidizing agents. A reducing agent is referred to an element which donates an electron to another chemical species in a redox chemical reaction. Since the reducing agent loses electrons, it is considered to be oxidized. Examples of such gases are SO_2_, H_2_S, H_2_, CO, NH_3_, and CH_4_. On the contrary, an oxidizing agent (or an electron acceptor) gains an electron in a chemical reaction. Examples of oxidizing agents include nitrogen oxides (NOx), oxygen, ozone, chlorine, fluorine, halogen gases, and nitric acid. In these cases, when an agent loses or accepts electrons, then the agent will be in lower or higher oxidation state, respectively.

The gas sensing mechanism of SiNWs is similar to the gas sensing mechanism reported for metal oxide semiconductors [36,44]. In the case of n-type semiconductors, the reaction with oxidizing gases decreases their conductivity, while reducing gases increase the conductivity (for p-type semiconductors it is opposite) [32,43].

Oxygen species have an important role in terms of the gas sensing properties of semiconductors since they can be adsorbed on the surface of the sensing layer, changing the acting mechanism of the sensor. The absorption of oxygen molecule to acting dangling bonds can be described through the following reactions [110]:

O_2(gas)_ → O_2(ads)_ O_2(ads)_ + e^−^ → O_2_^−^_(ads)_ O_2_^−^_(ads)_ + e^−^ → 2O^−^_(ads)_

The molecular oxygen ions, i.e., O_2_^−^ are stable below 150 °C, while atomic oxygen ions (O^−^ and O_2_^−^) are stable above 150 °C. Therefore, at the temperatures below 150 °C (suitable for SiNWs for proper operation), O_2_^−^ species are the predominant ions on the surface [110].

In ambient air, the absorption of oxygen ions on NW’s surface creates a hole accumulation layer (HAL) (in p-type SiNWs) or a depletion layer (in n-type SiNWs) by trapping electrons from the SiNWs [111].

**Figure 5:**
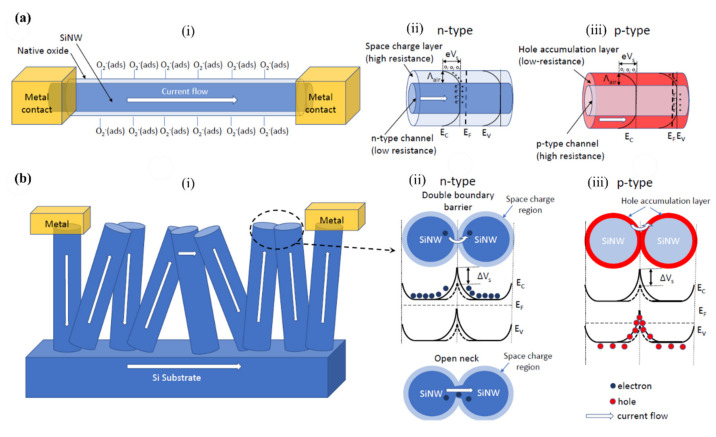
Schematic of (**a**)i a separate horizontal SiNW and (**a**)ii and (**a**)iii show conduction path in n-type (which is through inner part of SiNW) and p-type (which is through outer shell of SiNW) respectively. (**b**)i multiple vertical SiNWs with NW/NW junction barriers shown in (**b**)ii for n-type and (**b**)iii for p-type.

In principle, two kinds of configuration can be considered for SiNW: individual separated NWs where the electric current flows only along SiNWs, as shown in Figure 5ai, and a “zigzag” shape between the electrodes to form NW/NW junction, displayed in Figure 5bi.

In the first case, for the n-type SiNWs, the conductance depends directly on the diameter of conduction channel (Figure 5aii), and for the p-type SiNWs, it depends on the width of HAL (Figure 5aiii). A separation of SiNWs is often achieved by forming single NW or spaced multi NWs arrays, which are suspended between electrodes or be laid on dielectric substrate. In addition, the orientation of the SiNWs could be both horizontal or vertical with respect to the substrate.

In the zigzag (second) configuration, current flows through the connections between successive SiNWs as schematically presented in the Figure 5b. In this case the carriers have to overcome the surface potential barriers on the NW surfaces (for n-type) or are transferred directly through the HAL on the SiNWs surface (for p-type). It is schematically shown in the Figure 5bii,biii for n-type and p-type NWs, respectively. In many reports SiNWs are connected to the Si substrate where the current may flow alternatively through the substrate. Such connections may shorten the current path and, in some extent, aggravate the gas sensing properties of the structure. In order to solve this problem, an isolating layer (mainly of SiO_2_) can be deposited to separate the substrate from SiNWs. In addition, increasing the doping level in nanowires to enhance the conductivity of SiNWs can be alternative solution. In this particular case (Figure 5bi) we have well interconnected SiNWs. However, in the other case, we may have well-separated vertical nanowires, which in a way are somehow a vertical form of the first case (see Figure 5ai). It is also important to note that the multiple conductive paths (through the SiNWs, not the substrate) results in involving more SiNWs, which in turn results in more active sensing sites on each nanowire being involved in the gas sensing.

In order to sense a certain gas through SiNWs, there is a need for an interaction between gas molecules and SiNWs. This interaction can be the result of either direct absorption of gas molecules onto the surface of SiNWs (this can happen because of high electronegativity of gases) or the interaction between the gas molecules and molecular oxygen ions, i.e., O_2_^−^. It is apparent that, in some cases, both of these interactions can contribute to the sensing of the gas. This interaction, in the n-type SiNWs, can change the width of the depletion layer (and as a result the diameter of conduction channel), and in the p-type SiNWs can alter the width of the HAL and the surface potential value (V_s_), and finally the conductance properties of the SiNWs [111,112]. Therefore, it is interesting to investigate the different types of the gas (oxidizing and reducing gases) and their effects on the sensing mechanism in detail. Assuming that we have a p-type SiNW in vicinity of an oxidizing gas, this oxidizing gas extracts the electrons (which are minority carriers) from the conduction band in p-type Si and makes the HAL formed previously by oxygen ions, to become thicker. [111,112]. While the reducing gas releases the electrons trapped by O_2_^−^_(ads)_ and makes the HAL to become thinner. In terms of n-type SiNWs, the oxidizing gas extract electrons from conduction band in n-type Si and result in increasing the width of the depletion layer formed by oxygen ions, while the reducing gas decrease it by releasing trapped electrons. These changes in the HAL or depletion layer alter the conduction path and in reality, defines the sensitivity of the device.

Besides the chemical reactions, the physical adsorption (electrostatic or Van der Waals interactions) of gas or vapor molecules may also occur. In this case the polarity of absorbed molecules influences the surface potential of SiNWs. This kind of adsorption is crucial for humidity and volatile organic compounds (VOC) detection. For example, Cheng et al. [113] shows that polar molecules such as alcohols affect the SiNW conductance while the nonpolar substances like hexane do not affect them at all. On the other hand, in many cases the influence of polar molecules on electrical properties of semiconductor gas sensors causes poor selectivity towards humidity and other polar VOCs.

## 4. Resistors and Field Effect Transistors for Gas Sensing

The first, simplest, and most common configuration related to SiNW gas sensors is the resistor configuration. This sensor is based on detecting the conductance change in the SiNWs without the use of additional electric field from front gate or back gate [114]. Schematically, the resistance configuration may be considered, as shown in Figure 5. The electrical readout can be done by applying a DC or AC voltage to the electrical contacts (electrodes/metallization) and monitoring the current passing through SiNWs, or by direct measurement of the resistance by a sensitive ohmmeter. As described above the adsorption of the gas molecules onto the nanowires surface changes the conductance of the sensing structure, which changes the current or resistance output [115,116,117]. Gas concentration is indicated here by the amount of change in sensor resistance or current flow upon exposure to the gas molecules.

**Figure 6:**
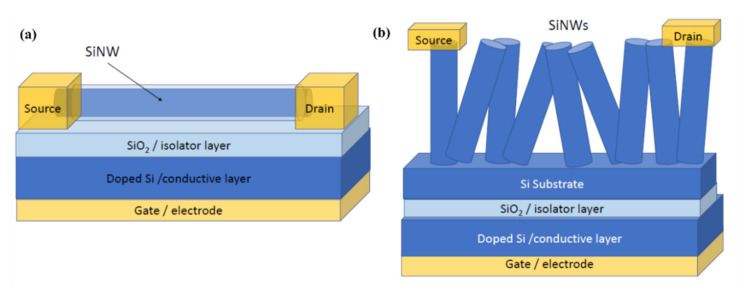
Schematic of SiNW-FET as gas sensors when NWs are formed (**a**) horizontally and (**b**) vertically.

Field effect transistors (FETs) are another common device group of gas sensors using SiNWs [43]. Since the SiNWs are formed on an insulating oxide layer (on SOI wafers), a back-gate configuration is usually formed for these transistors. In the case of the FET based configuration, SiNW functions as a conductive channel and this makes difference from conventional FETs [44]. The architecture of a horizontal and vertical SiNW based FET is shown in Figure 6a,b, respectively. In this configuration, SiNWs are connected to the two contacts known as source and drain. The number of charge carriers in the channel can be controlled by an electric field from gate electrode. For example, by applying a certain amount of gate voltage, SiNWs can be brought into depletion mode enabling one to measure in the subthreshold regime where the sensor is the most sensitive [118,119,120]. Doping is one of the important parts in the SiNWs that needs to be taken into account more seriously because it determines the number of carries inside the channel, and consequently the sensor’s sensitivity. In FET based sensors, we have the possibility to easily inject carries inside the channel by applying a constant voltage to the back-gate which is not possible in resistor-based sensors. Applying negative or positive voltage to the back-gate have different effect on the channel. Depending on the type of the channel (n-type or p-type), these negative or positive back-gate voltages can increase or decrease the number of carriers inside the channel. The sensing is performed by applying a constant voltage between the source and drain and monitoring the drain source current at a determined gate voltage. Even a few molecules of gases are sufficient to change the electrical conductance of channel and this signal will be enhanced due to the high surface to volume ratio of nanowires and gate effect of the FET amplifier configuration [121].

The electrostatically formed nanowire (EFN) sensor based on SiNWs is a multiple gate FET with silicon oxide surface that interacts directly with the target molecules and it is fabricated in a CMOS process, where the nanowire (conduction channel) is not defined physically but is electrostatically defined post fabrication and reduced to the nanometer size regime by controlling the surrounding gates. The EFN was firstly introduced in 2013 as a biosensor for real-time detection of femtomolar protein concentrations [122]. In some cases where machine learning is utilized, the selective detection is relying on the use of multiple parameters of the EFN sensor (threshold voltage (V_th_) and the drain-source on current (I_on_) for both junction and back gates). These sensor parameters are used as input for the training of the machine learning based classifier for the detection of the targeted gas [123]. The EFN gas sensor has two main advantages over other NW based gas sensors. The first one is related to the fabrication of EFN sensor that, using conventional silicon fabrication techniques with mature, relaxed, and well-developed design rules, results in low cost, robustness, and suitability for mass production. Second, the tunable size, shape, and even the location of the nanowire results in tunable sensing parameters, such as sensitivity, limit of detection, and dynamic range. The gas sensing properties of EFN based sensors are collected in the Table 2.

Basically, ionization gas sensors (IGS) and chemical gas sensors are two main approaches to detect molecules in gas phase. In recent years there were some reports utilizing SiNWs in IGS [124,125], however, they are not as common as chemical gas sensors, so we focus more on chemical gas sensors which are widely used in the electronics.

## 5. Impact of Functionalization on SiNWs Gas Sensing

### 5.1. Morphology and Size Effect

One of the efficient ways to improve the sensitivity and response-recovery characteristics of SiNWs is to increase the number of absorption sites on the surface of the nanowires. It is well known that the porous surface of SiNWs favors numerous surface defects and dangling bonds, which could effectively motivate the rapid adsorption of gas at room temperature, and thus longer SiNWs provide a much larger adsorption area for gas molecules [131].

**Figure 7:**
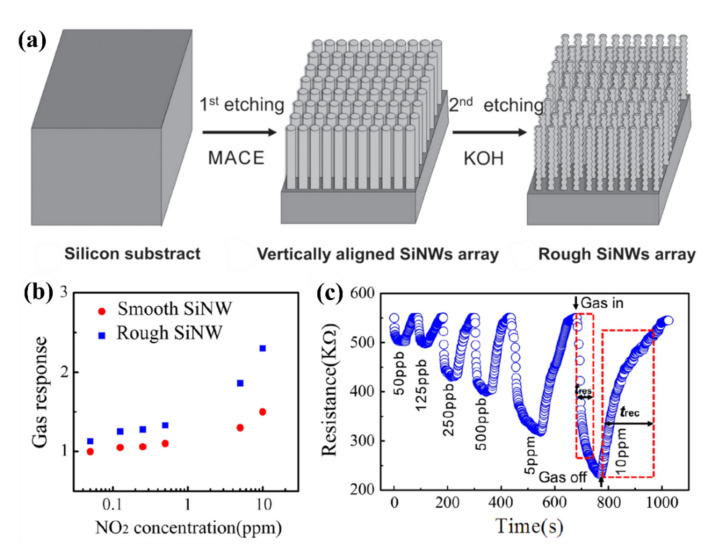
(**a**) Schematic illustration of the fabrication process for a rough SiNW array. (**b**) Sensor response as a function of NO_2_ concentration at room temperature for normal smooth SiNWs and rough SiNWs. (**c**) Dynamic response curve of the rough SiNWs array sensor to varying concentrations of NO_2_. Reproduced from [133], with permission from Springer Nature, 2020.

This can be achieved through changing the roughness of the surface of the SiNWs and creating more surface states for the absorption of gas molecules. For example, Y. Qin et al. [132] used the MACE technique to fabricate a smooth SiNWs array. Then, to further roughen the surface, this was followed by a KOH post-etching method. The post-etching time of KOH has an important influence on the surface roughness and thus on the sensing response of the SiNW sensor. The sensing response of the rough SiNW sensor to H_2_ is much superior to those of previously reported smooth SiNW arrays developed by the pure MACE process [132]. Figure 7a schematically shows the fabrication process of rough SiNWs for gas sensing purpose. In another report from this group, they used the same idea to increase the sensitivity towards detecting NO_2_ [133]. In this work, the rough SiNWs array due to KOH etching shows high active surface area and loose array configuration favorable for gas adsorption and rapid gas diffusion [133]. Figure 7b illustrates the gas sensor response as a function of NO_2_ concentration at room temperature for normal smooth and rough SiNWs. Also, Figure 7c shows dynamic response curve of the rough SiNWs array sensor to varying concentrations of NO_2_. As a result, the sensor based on rough SiNWs array is capable of NO_2_ detection with ppb level at room temperature, with good stability and satisfying response–recovery characteristics [133]. Since this configuration shows a good response to both H_2_ and NO_2_ gases, it cannot be considered as a selective way to detect these gases, however they have shown some selectivity study towards some special gases.

**Figure 8:**
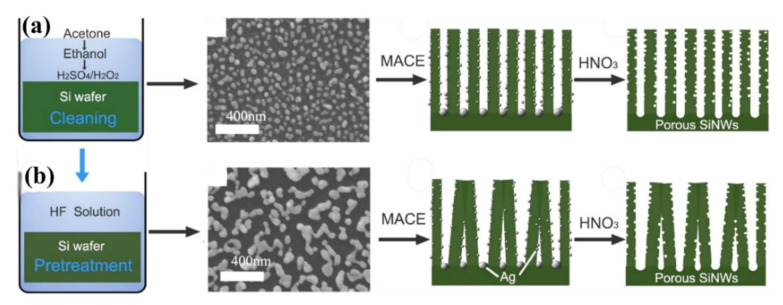
Schematic illustration of the etching models for the formation of (**a**) separating and (**b**) bundling SiNWs using MACE process. The SEM micrographs show in the part (**a**) uniform Ag nanoparticles formed on the untreated hydrophilic substrate and in the part (**b**) irregular Ag nanoflakes formed on the HF pretreated-induced hydrophobic substrate. Reproduced from [111], with permission from publisher John Wiley and Sons, 2020.

This group also investigated the fabrication of well separated vertical and bundling porous SiNW arrays by MACE method (see Figure 8a,b), based on the effective modulation of surface wettability of the initial Si substrate [111]. The HF pre-treatment creates a hydrophobic surface favorable for deposition of irregular Ag nanoflakes and then for the formation of bundling porous SiNWs array. In contrast, the porous SiNWs with well vertical separation are formed based on the pre-deposited uniform Ag nanoparticles on a hydrophilic Si surface. The porous SiNWs array featured by tip-clusters is proved to be highly effective in achieving highly sensitive and rapid response to NO_2_ gas at room temperature [111]. The attachment of the nanowires’ tips in the form of intercrossing between bunching clusters builds additional electrical conducting paths between electrodes during the gas-sensing measurement. The multiple conductive paths existing in the bundling of porous SiNWs sensor cause more SiNWs and more active sensing sites on each nanowire to be involved in the gas sensing [111]. The porosity of SiNWs and organization of the NWs next to each other (as a result of the nanowires’ tips attachment) are the main reasons for the improvement of gas response.

It is also noticed that changing a parameter during the process of NW growth, such as etching time (which effects the height of the NWs), can affect the sensing properties. Wang et al. proved that etching time has a great influence on the specific surface area of SiNWs, which will affect the gas sensing properties. The gas sensor based on the SiNWs exhibited a high gas response value and good selectivity to NO_2_ gas at room temperature [134].

**Figure 9:**
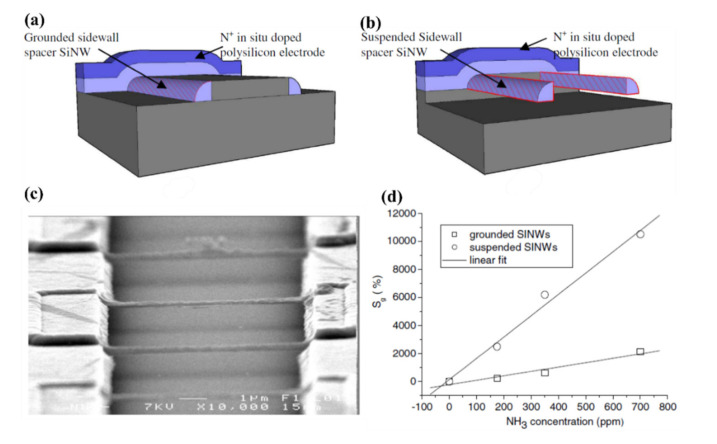
Schematic view of grounded (**a**) and suspended (**b**) sidewall spacer polycrystalline SiNWs. (**c**) SEM image of suspended polycrystalline SiNWs based sensing structure. (**d**) Relative response (S_g_ = (R_g_ − R)/R_g_) of the sensors vs. the ammonia concentration for both suspended and grounded SiNWs resistors. Reproduced from [135].

Other approach to morphological improvement of SiNWs sensors is application of suspended horizontal SiNWs proposed by Pichon et al. [135]. Authors presented here improvement of NH_3_ sensing properties by fabrication of the suspended undoped polysilicon NWs using wet etching of SiO_2_ on which previously NWs were obtained using plasma etching. The device is shown in the scheme and SEM image in the Figure 9a–c. The electrodes of the device were obtained by in-situ doping of the part of the device. As shown in Figure 9d, the suspended SiNWs were much more sensitive (relative sensitivity of 15.1%/ppm) to NH_3_ than grounded ones (relative sensitivity of 4%/ppm) in the same configuration. The authors claim that the reason for the sensitivity enhancement is the increase of the active area of the NWs.

**Figure 10:**
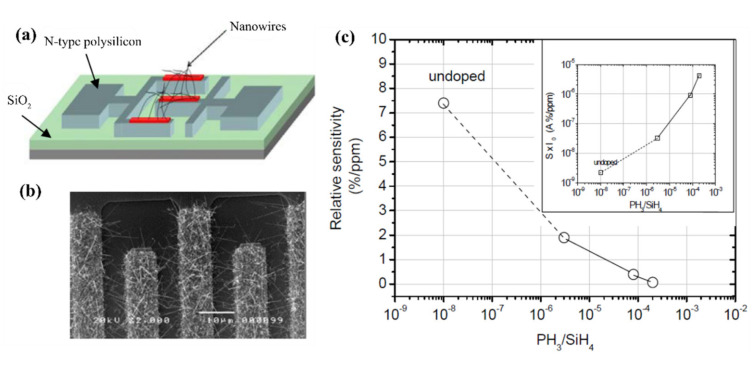
(**a**) Schematic view and (**b**) SEM image of the inter-digitated comb-shaped SiNWs based sensor. (**c**) Relative sensitivity to ammonia detection versus the phosphine to silane ratio (the insert shows the effect of the doping level on the sensitivity to ammonia detection molecules). Reproduced from [136], with permission from John Wiley and Sons, 2020.

There is a report from L. Pichon et al. which has investigated the n-type phosphorus doping effect on the sensing properties of SiNW for NH_3_ detection at room temperature [110]. In this work, the SiNWs were fabricated by VLS method using gold as catalyst. The SiNWs have inter-digitated comb-shaped structures (Figure 10a,b) fabricated in a 3-D configuration. As illustrated in Figure 10c, the study highlights that the relative sensitivity decreases, whereas the sensitivity increases, with the increase of the in-situ phosphorus doping level of the SiNWs.

The mechanism of this sensors is explained in two main theories: charge exchanging effect and chemical gating effect. The charge exchanging effect means that due to the reducing effect (electron donor) of ammonia the NH_3_ molecules adsorbed on the surface of the SiNWs could transfer charges. This phenomenon could directly inject electrons into the SiNWs, thus increasing the conductivity. Moreover, as SiNWs conductance can be modulated by an applied voltage, positively charged gas molecules bound on SiNWs surface can modulate their conductance by changing the volume of the conductive layer. In this case, the adsorbed gas molecules (NH_3_^+^) may act as chemical gates which shift the Fermi level of the SiNWs in the upper part of the band gap and reduce the resistance of the device. Table 3 summarizes the properties of bare SiNW gas sensors that we have discussed up to now.

### 5.2. Decoration by Metal Nanoparticles

**Figure 11:**
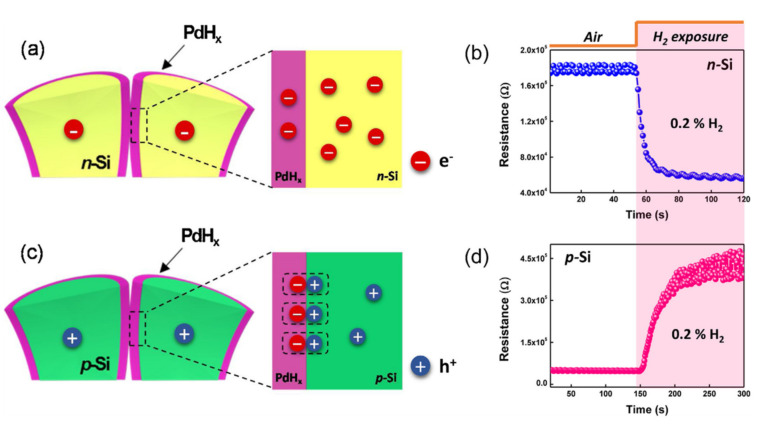
Schematic illustration of H_2_ sensing mechanisms in (**a**) n- and (**c**) p-type Pd-coated SiNW arrays based on carrier concentration. Resistance variation with time for 0.2% H_2_ depending on the major carrier types in (**b**) n- and (**d**) p-type Pd-coated Si NW arrays. Reproduced from [139], with permission from Elsevier, 2020.

Combining nanoparticles as catalyst with SiNWs can play a very important role in selective detection of gas molecules. For example, a selective response to H_2_ gas can be achieved by coating palladium (Pd) onto the surface of SiNWs [139]. It is well known in the literature that Pd is a good catalyst for more efficient hydrogen dissociation by considerably reducing the hydrogen adsorption activation energy. The mechanism is well shown in Figure 11 both for p- and n-type SiNWs [139]. As shown in Figure 11a in the case of n-type Si NW arrays, the dissociation of hydrogen molecules into hydrogen atoms converts the coated Pd on SiNWs to palladium hydride (PdH_x_), which lowers the work function of Pd, thereby facilitating the transfer of electrons from PdH_x_ to n-type SiNWs [139].

**Figure 12:**
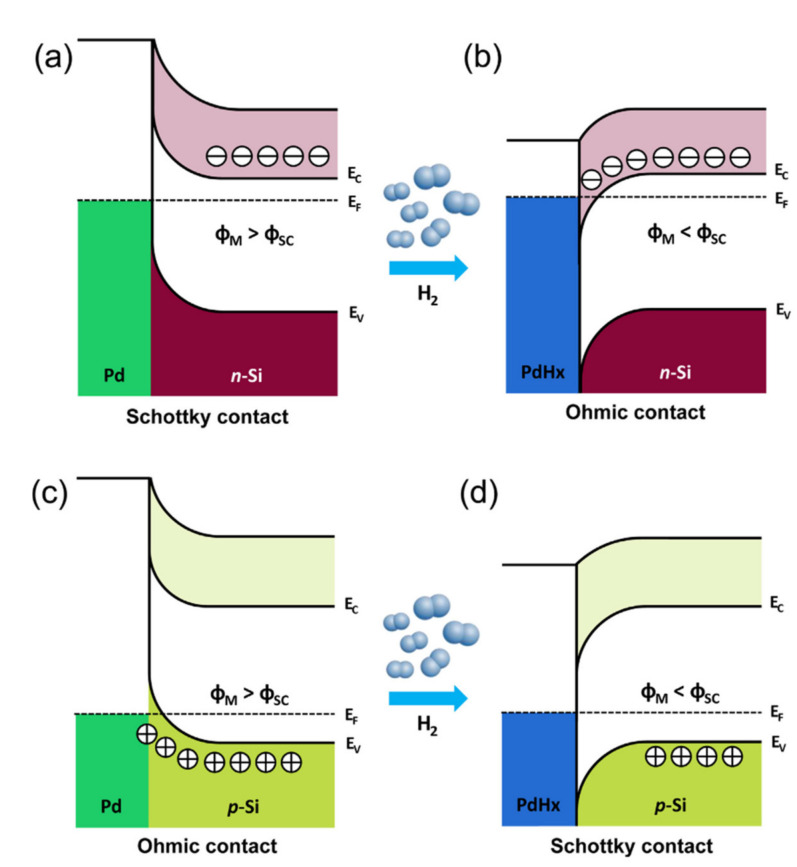
Schematic illustration of the change in contact resistance at the metal (Pd)-semiconductor (Si) junction: (**a**) formation of Schottky barrier in an n-type SiNW before the exposure of H_2_, (**b**) formation of Ohmic contact in the n-type SiNW after the exposure of H_2_, (**c**) formation of Ohmic contact in the p-type SiNW before the exposure of H_2_, and (**d**) formation of Schottky barrier in the p-type SiNW after the exposure of H_2._ Reproduced from [139], with permission from Elsevier, 2020.

In other words, upon exposure to H_2_, the resistance of the Pd-coated n-type SiNW arrays decreases, as shown in Figure 11b. In fact, since the work function of Pd is larger than that of Si, a Schottky barrier is formed between Pd and n-type SiNW before exposure (Figure 12a). After exposure to H_2_, an Ohmic contact is formed due to the reduction of work function owing to the formation of PdH_x_ (Figure 12b). In the case of p-type SiNWs, when exposed to H_2_, we have the same reduction in the work function as a result of PdH_x_ formation (Figure 11c,d). This can facilitate the transfer of electrons to the p-type SiNWs, which neutralizes the hole carriers (see Figure 12c). Thus, the resistance of the Pd-coated p-type SiNW arrays increases (see Figure 11d). In this case, before exposure, we have an Ohmic contact between Pd and p-type SiNW (see Figure 12c) which changes to a Schottky contact upon exposure to H_2_ due to the reduction of Pd work function (see Figure 12d) [139]. As a result of a decrease in the work function of PdHx, with increasing H_2_ concentration, the height of the Schottky barrier increases. However, since the barrier in the n-type SiNW arrays changes to an Ohmic contact upon exposure to H_2_, the interface effect of Pd/Si diminishes with increasing H_2_ concentration. Consequently, the sensitivity of the Pd-coated p-type SiNW arrays is much higher than that of the n-type NW arrays. A native SiO_2_ layer in Pd/Si interface serves as a diffusion barrier against palladium silicide (PdSi) formation while concurrently reducing the effect of Fermi level pinning. If the SiO_2_ layer is not formed on the n- or p-type SiNW, a Schottky barrier forms between PdSi and SiNW, resulting in no response to hydrogen gas [139].

**Figure 13:**
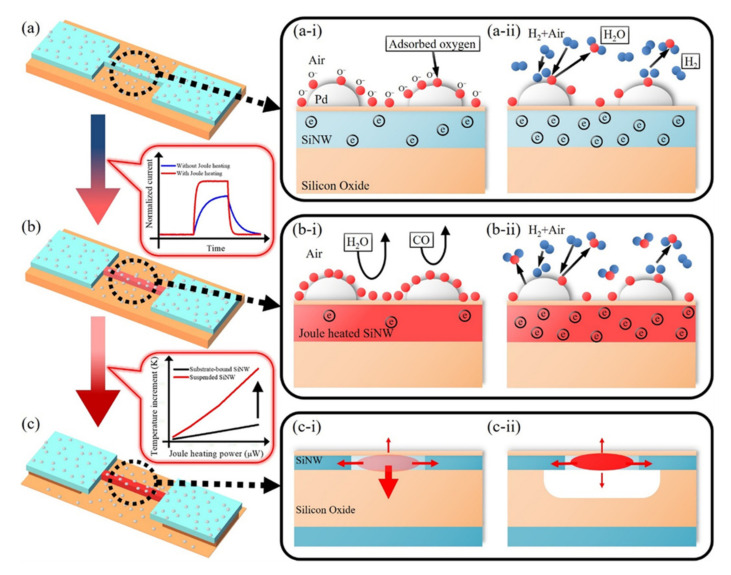
Working principle of H_2_ sensing of Pd-SiNWs: (**a**) at room temperature, (**a**-**i**) depletion of charge carrier (electron) in SiNW (n-type) by negatively charged adsorbed oxygens (red dots) and (**a**-**ii**) accumulation of charge carrier by desorbing oxygen with H_2_O formation under H_2_ gas exposure; (**b**) Faster and higher response with self-heating of Pd-SiNW because of (**b**-**i**) more depletion of charge carrier due to more adsorbed oxygen and (**b**-**ii**) fast reaction rate with H_2_; Low interfering gas effect (H_2_O and CO) with self-heating; (**c**) Lowered power consumption by reducing heat loss through the substrate by changing from substrate-bound SiNW to suspended SiNW. Reproduced from [141], with permission from American Chemical Society, 2019.

**Figure 14:**
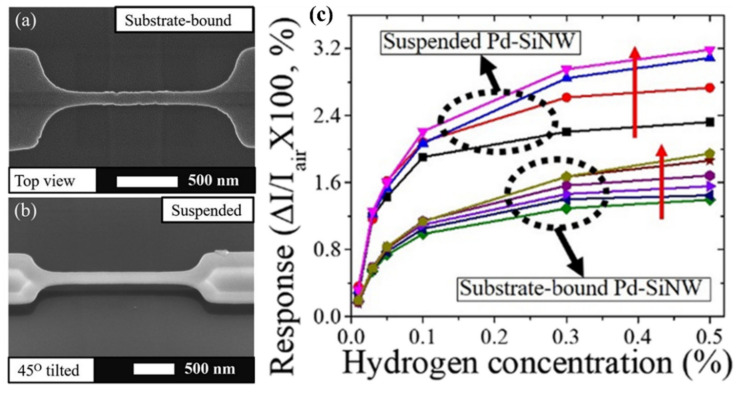
SEM images of a (**a**) substrate bound, and (**b**) suspended SiNW. A comparison between the substrate-bound and suspended Pd-SiNW sensors is shown in (**c**) showing responses with various self-heating powers (red arrows: direction of self-heating power increment (from 41 to 147 μW for the suspended Pd-SiNW and from 205 to 1172 μW for the substrate-bound Pd-SiNW)). Reproduced from [141], with permission from American Chemical Society, 2019.

Several articles have investigated the effect of Pd nanoparticles as catalyst on the surface of SiNWs for H_2_ detection [140,141,142,143,144]. The studies demonstrate that the combination of Pd nanoparticles, self-heating as well as suspension structure lead to an enhancement of the gas sensing properties of Pd-SiNWs. The results show that suspended Pd-SiNWs (fabricated by using conventional CMOS-compatible processes like deep ultraviolet lithography, oxygen plasma, reactive ion etching, ion implantation and rapid thermal annealing) are excellent H_2_ sensor with fast response and recovery time (due to the self-heating effect). Such sensors operate at sub-milliwatt power and have H_2_ detection characteristics which are comparable to those of the substrate-bound Pd-SiNW at much lower operation power [141]. The schematics in Figure 13 show the working principle of H_2_ sensing of a Pd-SiNW at room temperature and elevated temperature. The oxygen adsorption effect for H_2_ response of Pd-SiNW was adopted to understand the results of increased response with self-heating of Pd-SiNW. In addition, the self-heating of Pd-SiNW was found to reduce the influence of interfering gases like humidity and CO on the sensing characteristics to H_2_ gas [141]. Figure 14a,b depicts the SEM micrographs of substrate bound and suspended SiNW, respectively. A comparison between the results of these two configurations is shown in Figure 14c, where the response verses H_2_ concentration for different self-heating powers is presented [141].

An additional report related to the detection of H_2_ by Pd nanoparticles is presented in [145]. In another similar work, SiNWs were modified with nanoparticles of Ag, Au, Pt and Pd using MACE method for room temperature H_2_ detection [146]. It is demonstrated that the modification considerably improves the response of the sensor especially in the case of Pt. However, the modification with Ag and Au gives fast time of response and recovery for low and high H_2_ concentrations respectively. The response of Ag and Pd modified structures is observed for high H_2_ concentrations (more than 85 ppm) [146]. Hassan et al. utilizes Pt-Pd for its better hydrogenation property in comparison to pure Pd. At higher temperatures (temperatures above 100 °C), Pt is considered a superior catalyst for hydrogenation reaction, which is the rate limiting reaction for a sensor response [147].

Kim et al. demonstrated the NH_3_ sensing characteristics of SiNW FETs with AuNPs decoration to enhance the sensitivity and long-term stability [148]. The operation in the subthreshold regime provides higher sensitivity, lower power consumption, and sufficient linearity. The decoration of the SiNW surface with AuNPs is an effective method to realize nanowire FET-type sensors with high sensitivity and high reliability for chemical sensing applications [148]. The sensing mechanism is the same as what we have discussed before for PdNPs [148]. It is also worth noting that Au modified SiNWs used to detect CO_2_ [149].

**Figure 15:**
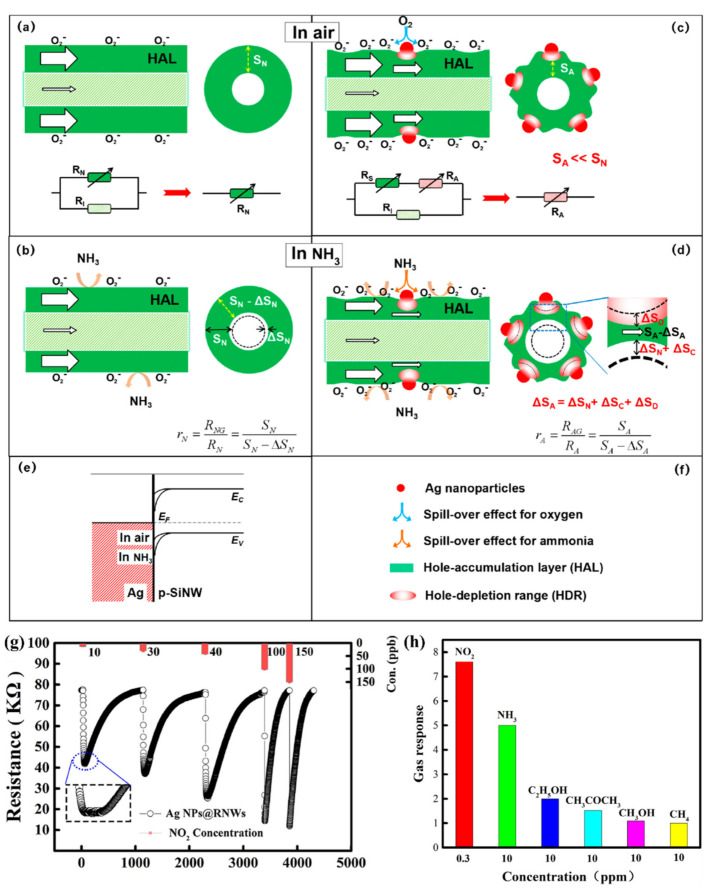
Schematic illustration of gas sensing mechanism of (**a**,**b**) bare p-SiNW and (**c**–**e**) Ag modified rough p-SiNW sensor, (**f**) the corresponding description of symbols, (**g**) dynamic response curves of the sensors based on Ag NPs@RNWs to varying concentration of NO_2_ at room temperature and (**h**) response of the Ag NPs@RNWs sensor to different gases: the concentration of NO_2_ at 0.3 ppm and others at 10 ppm. Reproduced from [150] and [151], with permission from American Chemical Society, 2017 and Elsevier, 2020.

It has been noticed in a series of reports about the nanoparticles such as Ag deposited onto the SiNWs to detect NO_2_ and NH_3_ [150,151]. For example, Y. Qin and et al. developed a novel and cost-effective process to prepare Ag-modified SiNW sensors and further suggested a resistance effect model to clarify the enhanced sensing mechanism of Ag-modified SiNWs towards NH_3_ [150]. The crucial procedure of tetramethyl ammonium hydroxide (TMAH) post etching forms a loose array of SiNWs with rough surface (RNWs) favorable for rapid diffusion and adsorption of gas molecules. It is expected that the redistribution of Ag nanoparticles is important to form highly discrete and firmly attached tiny Ag nanoparticles on the rough surface of the nanowires [150]. They could justify the sensing of NH_3_ through a resistance effect model presented in Figure 15. For bare SiNW (Figure 15a), after forming HAL due to the adsorbed molecules, mainly oxygen and water, from the atmosphere, we have two resistance in parallel (one for HAL (R_N_) and the other for inner part of SiNW (R_I_)). The much smaller resistance R_N_ dominates the conduction of the p-type SiNW. The cross sectional area (S) of the HAL shell determines the resistance of the nanowires by $R=\frac{\rho L}{S}$, where L is nanowire length, ρ is resistivity, and S is the cross sectional area of the HAL shell. When the Ag nanoparticles attached on the surface of SiNW, due to the difference in work function between them, the transfer of electrons occurs from AgNPs to p-SiNWs at the interfaces as shown in Figure 15e. As a result, we will have small hole depletion regions around the AgNPs according to Figure 15c. These regions decrease the cross-section area and create R_A_ in series with the previous normal resistance (R_S_). In this case R_A_ dominates the total resistance of the nanowire. Upon exposure to NH_3_ gas, the adsorbed NH_3_ molecules will inject electrons into the HAL shell through direct and indirect ways, due to the reducing effect (electron donor) of ammonia. The effective injection of electrons results in an obvious shrinkage of HAL, as illustrated in Figure 15b,d. Consequently, the resistances of both the bare SiNWs and the Ag-SiNWs increase.

A similar method has been also applied for the detection of an oxidizing gas, namely NO_2_ [151]. The results are presented in Figure 15g,h. Ag modified SiNWs showed good selectivity towards NO_2_ gas among some other interfering gases (Figure 15h) [151].

Hsu et al. formed Ni-silicide nanocrystal on p-type SiNW for O_2_ sensing (SiNWs were fabricated by atomic force microscope nano-oxidation on SOI substrate, selective wet etching, and reactive deposition epitaxy)[152]. The change in current in Ni-silicide/SiNW increases after the exposure of the nanowire to O_2_. This phenomenon can be explained by the formation of a Schottky junction at the Ni-silicide/Si interface in the Ni-Silicide/Si nanowires and the formation of a hole channel at the silicon nanowire/native oxide interface after exposing nanowires to O_2_ [152].

There is also a similar work used Ni for surface modification to detect Cl_2_ [153]. The authors have demonstrated the CVD growth of SiNWs, as well as the assembly of Ni-Si NWs on molecularly patterned substrates, and their application to sensors for the detection of Cl_2_ gas. The Ni-Si NWs have a larger surface-to-volume ratio compared to that of Ni NWs, which makes them more advantageous in detecting Cl_2_ gas. The Ni-Si NW sensor showed the real-time detection of Cl_2_ gas with high sensitivity and fast response time [153]. Table 4 presents the recent papers utilizing SiNWs gas sensors functionalized by NPs for detecting different gases.

### 5.3. Doped Junctions

#### 5.3.1. Homojunctions

Si has a huge potential because of easy way of obtaining both n- and p-type structures by well-established doping methods. This creates an opportunity of obtaining both n- and p-type SiNWs and thanks to that creation of homojunctions.

**Figure 16:**
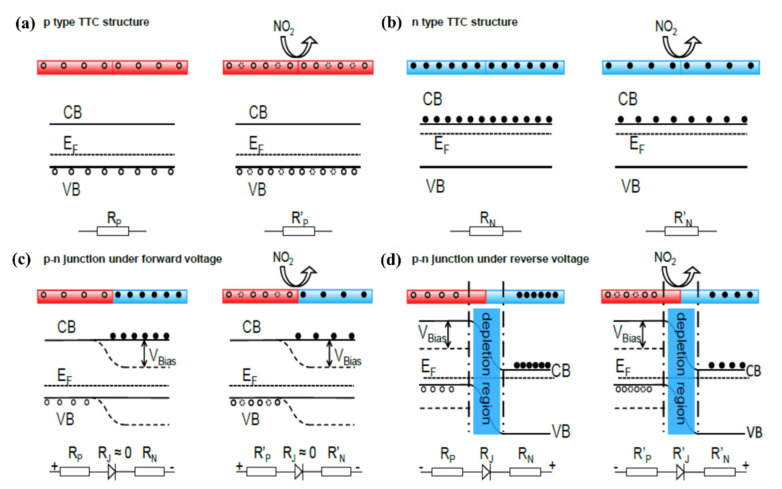
Schematics and energy band diagrams of different contact structures before and after being exposed to NO_2_ for (**a**) p-type SiNWs contact structure, (**b**) n-type SiNWs contact structure, p-n homojunction under forward voltage (**c**) and reverse voltage (**d**). Reproduced from [137], with permission from RSC Pub, 2020. (●, electron; ○, hole).

Lin et al. have demonstrated that vertical SiNWs array can be jointed with each other at the tip ends by joule heating treatment to form nanowires with p-p (both sides are p-type) and n-n (both sides are n-type) contacts as well as p-n junction for gas sensing purpose [137]. This structure not only resolved the problem of electrode contact encountered in common nanowire sensors, but also elongates the nanowire length to produce sensitive response to NO_2_ at ppb level at room temperature [137]. Figure 16 shows the gas sensing mechanism before and after exposure to NO_2_ for the SiNWs with p-p contact (Figure 16a), n-n contact (Figure 16b), p-n junction under forward bias (Figure 16c) and p-n junction under reverse bias (Figure 16d). It is apparent that, for both the p-p and the n-n contact after Joule heating, they become normal p- and n-type SiNWs and the mechanism is the same as what we discussed earlier. It is interesting to mention that the response of p-n tip-tip contact SiNW array under the forward bias, as shown in Figure 16c, is insignificant because of the opposite response on p- and n-type semiconductor. Meanwhile, under the reverse bias, the p-n junction displayed a significant rectification effect, and by monitoring the reverse current that originated from electrons (minority carriers of p-type SiNWs) in the presence and absence of target gas, a reliable sensor with a new structure can be achieved [137].

#### 5.3.2. Heterojunctions with Inorganic Semiconductors

It is also worth investigating the functionalization of SiNWs by metal-oxide (MOX) semiconductors, e.g., ZnO, SnO_2_, TiO_2_, WO_3_ which are the most popular gas sensitive materials. These materials are highly sensitive to many gases and vapors, have good long-term stability and their fabrication is cost-effective. The major problems to be solved for MOX based gas sensors are their requirement for operation at high temperatures and poor selectivity. There is a large interest to create heterojunctions between different semiconductor nanostructures via materials mixing, growing shell-core structures, creating multilayer structures, etc., for improving the gas sensing properties. In latest years, several approaches in this field were adopted using porous Si as a conducting substrate for MOXs based nanostructures [154,155].

**Figure 17:**
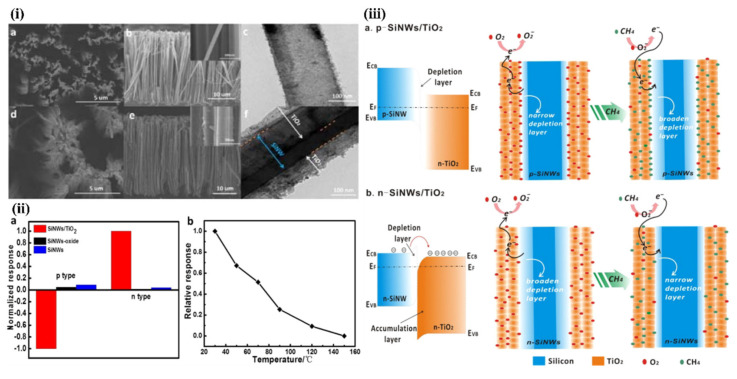
SiNWs/TiO_2_ core-shell structures for CH_4_ sensing: (**i**) SEM images of SiNWs before (**a**,**b**) and after (**d**,**e**) TiO_2_ deposition and TEM images of SiNW (**c**) and SiNW/TiO_2_ (**f**) structures; (**ii**) (**a**) n- and p-type SiNWs based sensors (bare, thermal oxidized and TiO_2_ coated) responses to 100 ppm of CH _4_ at RT, (**b**) the conductive response of n-SiNWs/TiO_2_ sensor to 100 ppm of CH_4_ at different temperatures.; (**iii**) schemes of RT CH_4_ sensing mechanism for (**a**) p-SiNWs/TiO_2_, (**b**) n-SiNWs/TiO_2_ Reproduced from [156], with permission from American Chemical Society, 2017.

Liu et al. presented gas sensor based on SiNW/TiO_2_ core-shell heterojunctions for methane sensing [156]. In this work, vertical SiNWs array was fabricated using MACE method and then coated by TiO_2_ using sol-gel method. As can be observed from the SEM and TEM images in the Figure 17i, the SiNWs are slightly bent and they consist a congregated bundle structure with a coating of 100 nm TiO_2_ layer over the SiNWs with 35 µm in length and 100–200 nm in diameter. The authors compared the sensing properties of bare SiNWs, thermally oxidized SiNWs and SiNWs-TiO_2_ heterostructures for both n- and p-type SiNWs (Figure 17ii) and showed the high impact of TiO_2_ to the CH_4_ sensing properties. SiNWs are serving here as a main conduction path while TiO_2_ serves as gas sensitive medium. Authors proposed here the possible sensing mechanisms for both n- and p-type SiNWs and n-type TiO_2_ and showed it schematically in the Figure 17iii. The outcome of this study shows that p-type SiNWs and TiO_2_ create a p-n junction at the interface, and because of differences in the Fermi level between these materials, charge carrier diffusion occurs, resulting in the formation of a depletion layer. Size of this depletion layer is determined by inner TiO_2_ electric field which is depending on the quantity of electron taking O_2_^−^ adsorbed on TiO_2_. So, in this case the depletion layer is narrow at the air conditions and the CH_4_ acts here as a reducing gas which caused the release of some of electrons trapped by oxygen. This leads to increase the depletion layer and finally limited the current flow through the structure (p-type response to reducing gases). For n-type SiNWs/TiO_2_, the n-n heterojunction is created, and thanks to possible electron transfer from SiNW to TiO_2_ the depletion layer is created in the SiNW surface. In this case the adsorption of O_2_^−^ increases the depletion layer (more electrons are taking from SiNW). The reducing reaction (because of CH_4_) in this case leads to decrease in depletion layer and finally to increase of the current flowing through the structure (n-type ration). The proposed sensor is operating at room temperature and leads to very low power consumption (only 1 V of supply voltage and µW level power consumption). The sensor has a detection limit of 20 ppm of CH_4_ (with confirmed linear response in the range of 30–120 ppm) [156]. However, this sensor, as many other common MOX based sensors, is limited by the influence of humidity to the responses, poor selectivity (responses to ethanol and acetone vapors are even higher than to CH_4_, n-type SiNWs based structure is sensitive even to changes of N_2_ level in the air) and strong response dependence on the operating temperature.

**Figure 18:**
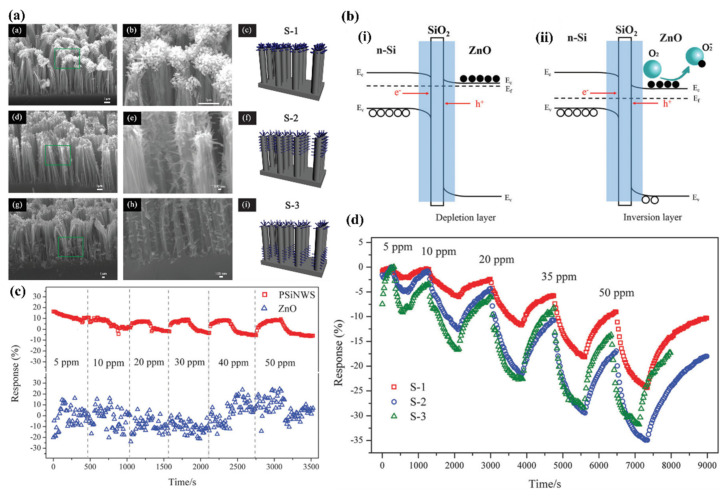
(**a**) SEM images of ZnONWs/PSiNWs hybrid strictures and schemes of these structures with different ZnO coverage; (**b**) scheme of proposed sensing mechanism-energetic bands of ZnONWs/PSiNWs (**i**) before and (**ii**) after exposure to oxidizing gas; (**c**) response of the of bare PSiNWs and ZnO to NO_2_ at RT; (**d**) responses of ZnONWs/PSiNWs hybrids presented in SEM images. Reproduced from [157], with permission from Royal Society of Chemistry, 2020.

Liao et al. presented a porous SiNWs/ZnO NWs hybrid for NO_2_ sensing at RT [157]. The work presented the structures of n-type PSiNWs, obtained by Ag-MACE, covered by ZnO nanowires grown by the hydrothermal method. Three structures of ZnO nanowire/PSiNWs (Figure 18a) differed by the level and place of coverage of PSiNWs by ZnO NWs. These structures obtained by different preparations of the substrate (different distribution of crystallite spores on wafer with PSiNWs) were investigated. NO_2_ sensing properties of these structures (Figure 18d) were compared to bare PSiNWs and ZnO NWs (Figure 18c), respectively. In all cases, ZnO/PSiNWs hybrids were more sensitive to the NO_2_ than bare materials and the responses were also dependent on the level of coverage of PSiNWs by ZnO NWs. Interestingly, while two n-type material heterojunctions were formed, the gas sensing behavior for the oxidizing gas is typical for a p-type semiconductor (resistance decreases after reaction with NO_2_). The authors explained it by the energy levels fitting on the ZnO/SiNW interface and the differences in electron affinity. As shown in the energy band diagrams (Figure 18b), before reaction to oxidizing gas, the depletion layer is created because electrons from SiNW are transported to ZnO resulting holes to transport from ZnO to SiNW. The oxygen adsorbed from air captures electrons from the ZnO and holes becomes a major charge carrier in the interface region, as the inversion layer is created. Exposure to NO_2_ is causing stronger oxidation than in the clean air thanks to that the holes concentration in inversion layer increases and as a result resistance decreases. This type of sensor is much more sensitive to NO_2_ than NO, NH_4_, and methanol. They also observed the sensor recovery process after reaction to NO_2_ at RT [157]. However, the values of sensor responses for relatively high NO_2_ concentration (5–50 ppm) reported here are relatively low, as sensor response time and recovery time are both slow and a significant baseline drift is observed. This shows that this concept needs to be improved.

**Figure 19:**
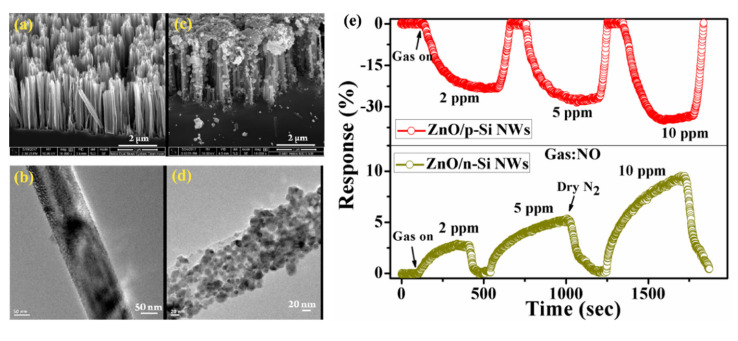
SEM and TEM images of SiNWs before (**a**,**b**) and after (**c**,**d**) ZnO deposition; (**e**) response of SiNWs/ZnO heterojunction to NO at RT in N_2_ atmosphere for both n and p-type SiNWs. Reproduced from [158], with permission from IOP Publishing, 2020.

Next approach to ZnO/SiNWs heterojunction was proposed by Ch. Samanta et al. [158] for detection of low concentrations of NO at RT. The SiNWs array was grown similarly as in previous example by Ag-MACE method and then the ZnO layer was deposited by the chemical solution deposition method. In this case the ZnO morphology on SiNWs is found to be a nanograin film (Figure 19a–d). The authors made and compared structures based on both n- and p-type SiNWs. As shown in Figure 19e, the responses to NO had n-type character for n-type Si and p-type character for p-type Si. The authors found that the use of p-type SiNWs gives a higher and faster response to NO at a concentration range of 2–10 ppm. However, it needs to be stressed that in this case experiments were carried in oxygen free atmosphere (N_2_) and it is hard to compare them with the previous example.

**Figure 20:**
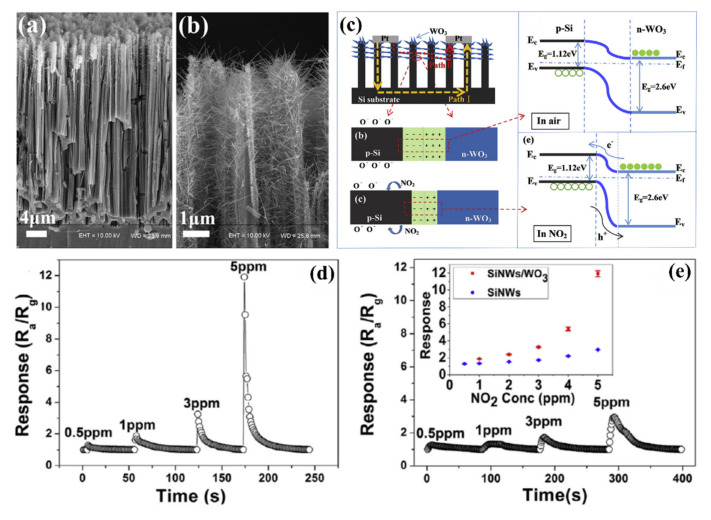
(**a**,**b**) The side view SEM images of SiNWs/WO_3_ nanowires. (**c**) Schematic illustration for gas-sensing mechanism of SiNWs/WO_3_ sensor, structural model, and heterostructure models and energy band diagrams in air and in NO_2_. Dynamic responses of the composite (**d**) and the pure SiNWs (**e**) to 0.5–5 ppm NO_2_ at RT. Reproduced from [159], with permission from Elsevier, 2020.

SiNWs/WO_3_ NWs composite with dendric morphology for RT NO_2_ sensing was reported by Y. Qin et al. [159]. The p-type SiNWs array was also produced by Ag-MACE method and the WO_3_ nanowires were grown using thermal oxidation of the W film, which had been pre-deposited using magnetron sputtering. Thanks to that on the top part of well separated SiNWs, WO_3_ NWs, creating connections between SiNWs which are similar to the treetops, were grown (Figure 20a,b). The structure, with possible current flow paths, is schematically presented in the Figure 20c. Therefore, these energy band diagrams of the SiNW/WO_3_NW interface before and after exposure to NO_2_ explains the sensing mechanism. In this case, the current is flowing via the SiNW/WO_3_ junctions (Path II) rather than via interconnections between SiNW or substrate. This is why the size of the depletion layer in the interface is crucial to determine electrical properties of the structure. The authors explain here, that NO_2_ molecules interact with SiNWs and extract the electrons from SiNWs surface as a result increases the number of holes. This leads to change the balance in the depletion region because of the transfer of electrons from WO_3_ to Si region that decreases the depletion region size and as a result decreases the structure resistance. Proposed sensing structure offers high and very fast response (less than 1 s) to NO_2_ in the concentration range of 0.5–5 ppm at RT. As can be seen in Figure 20d,e, SiNWs/WO_3_NW structure sensing properties are significantly better than sensing properties of bare SiNW. The same group also presented a similar, so-called cactus-like SiNWs/WO_3_ structure [160] where the enhancement of NO_2_ sensing properties was also reported.

**Figure 21:**
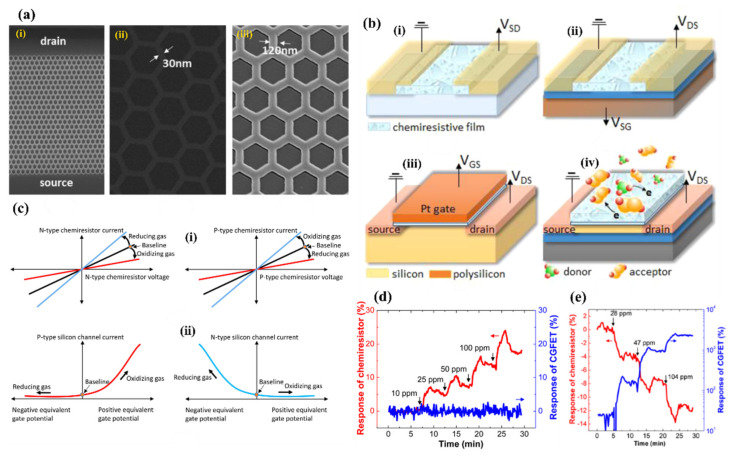
(**a**) SEM images of the (**i**) fabricated SnO_2_ CGFET, (**ii**) close-up view of the honeycomb channel region before and (**iii**) after the SnO_2_ deposition process, (**b**) schematic illustration of various gas sensor structures: (**i**) two terminal chemiresistor, (**ii**) back-gated FET, (**iii**) platinum gate FET typically used as hydrogen sensors, and (**iv**) metal-oxide floating gate CGFET. (**c**) Conceptual illustration of the response of (**i**) chemiresistor and (**ii**) CGFET for oxidizing and reducing gases. The monotonic function of the chemiresistor results in a response to both oxidizing and reducing gases. The nonlinearity of the normally off CGFET selectively responds to the corresponding type of gas. The response of the CGFET and the control chemiresistor with different concentration for (**d**) ammonia and (**e**) O_2._ Reproduced from [161], with permission from American Chemical Society, 2020.

In 2015, Han et al. [161] presented honeycomb-like structure of 1d SiNW matrix (fabricated by conventional top-down technology including lithography and plasma etching) coated by SnO_2_ (using sputtering method) as a gas sensing structure in both transistor and resistor configurations. Figure 21a shows the SEM images of these honeycomb structures. The SiNWs serve here as a conduction channel while the SnO_2_ film on its top servs as a gas sensitive medium. This type of sensor, which is called a chemically gated field effect transistor (CGFET), is important because of the operation of sensor with low drive voltage and high reliability [161]. The potential change induced by the molecular adsorption and desorption allows the electrically floating sensitive material to gate the silicon channel [161]. As the device is designed to be normally off, the power is consumed only during the gas sensing occurrence. This feature is attractive for battery-operated sensors and wearable electronics with driving voltage of 1 V or below. In addition, the decoupling of the chemical reaction and the current conduction regions allows the gas sensitive material to be free from electrical stress, thus increasing reliability [161]. The concept of this type of sensor schematically compared with other types in Figure 21b. This normally off CGFET distinguishes between the oxidizing and reducing gases due to the nonlinearity of the channel as depicted in Figure 21c. For example, in order for the sensor to only respond to oxidizing gases, the n-channel device (p-type silicon) is preferred [161]. The authors also compare the CGFET and the control chemiresistor, and proved the different effect of reducing (NH_3_) and oxidizing gas (O_2_) on the sensor the result of which is presented in Figure 21d,e. The results clearly show differences between CGFET and resistor configurations. For p-type channel the response to NH_3_ is noticeable for resistor while the CGFET is not sensitive at all. In the case of oxidizing gases (O_2_ but also NO) the CGFET configuration is two orders of magnitude more sensitive than chemiresistor one. For oxidizing gases, the direction of the response for considered configurations is also different (for resistor conductivity decreases while for CGFET current flowing via structure increases). The explanation of this behavior for resistor configuration is based on the electron depletion of SnO_2_ layer caused by its oxidation since, here, the charge exchange on the current conduction channel occurs itself, the overall number of charge carriers decreases, resulting in the conductivity drop. In the case of CGFET, the pseudo positive potential in the gate creates the inversion channel in the p-type silicon NWs and increases their conductance.

**Figure 22:**
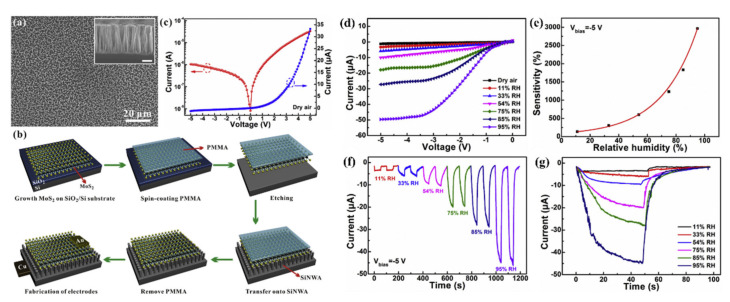
(**a**) SEM top and cross-section images of the SiNWs (The inset’s scale-bar is 2 μm). (**b**) the schematic diagram of process flow for a MoS_2_/SiNW heterojunction device, (**c**) I-V curves of MoS_2_/SiNW heterojunction in dry air, (**d**) I-V curves of MoS_2_/SiNW heterojunction at reverse voltage under varied RH values, (**e**) the dependence relation between sensitivity and relative humidity. (**f**) current response of MoS_2_/SiNW heterojunction to dynamic switches between dry air and varied RH valu,s at V_bias_ = −5 V, and (**g**) single-cycle response with different RH values. Reproduced from [162], with permission from Elsevier, 2020.

Beside the junctions with MOX wide bandgap semiconductors, attempts of creating heterojunctions of SiNWs with 2D materials, like graphene or MoS_2_, for gas sensing purpose were studied. The junctions with carbon materials, including graphene, are described in another subsection and here we are concentrating on the MoS_2_. For example, MoS_2_/SiNWs heterojunctions have been applied for relative humidity (RH) [162] and NO [163] sensing at RT. In these articles, on the top of vertical aligned n-type SiNWs (obtained by Ag-MACE shown in Figure 22a), MoS_2_ thin films (obtained by two step thermal decomposition process) were placed (transferred) using a PMMA-assisted method (Figure 22b). The I-V characteristics of this structure is shown in Figure 22c confirming the Schottky contact. It has been shown in ref. [162] that MoS_2_/SiNWs heterojunction is more sensitive to RH and its response/recovery times are shorter for reverse voltage than for forward one. The relative results are presented in Figure 22d–g for reverse bias at different humidity levels. As authors explained the H_2_O molecules are physically absorbed on the surface of MoS_2_ film and then free electrons are injected to the film thanks to Grotthuss chain reaction mechanism. As reverse current value is very sensitive to the changes of potential barrier width and height of n-MoS_2_/n-SiNW heterojunction the presence of humidity in the atmosphere causes significant increase in the current flow. For both reversal and forward voltages sensing RH properties of present structure are attractive for potential applications (at 95% RH for voltage bias of −5V: response = 2967% and response/recovery times = 22.2/11.5 s and for +5 V: 392% and: 26.4/15.1 s, respectively). The NO sensing properties of the same structure [163] are also better for reverse voltage bias, where authors obtained high sensitivity (response of 3518% @ 50 ppm), for wide concentration range (50–1000 ppm) of NO, and low detection limit (10 ppb). In this case, the NO molecules are interacting with the oxygen species adsorbed on the MoS_2_ and as a result release the electrons to MoS_2_ conduction band that causes the structure’s resistance to decrease. This study demonstrates the influence of RH on NO sensing properties of these sensors and shows that the maximum response is observed for RH value about 60%, however the structure still can operate at wide RH range. The structure shows good selectivity behavior with respect to other oxidizing gases like NO_2_ and O_2_. However, in this case, both structure’s response and regeneration times were relatively slow (both higher than 10 min @ 50 ppm of NO).

**Figure 23:**
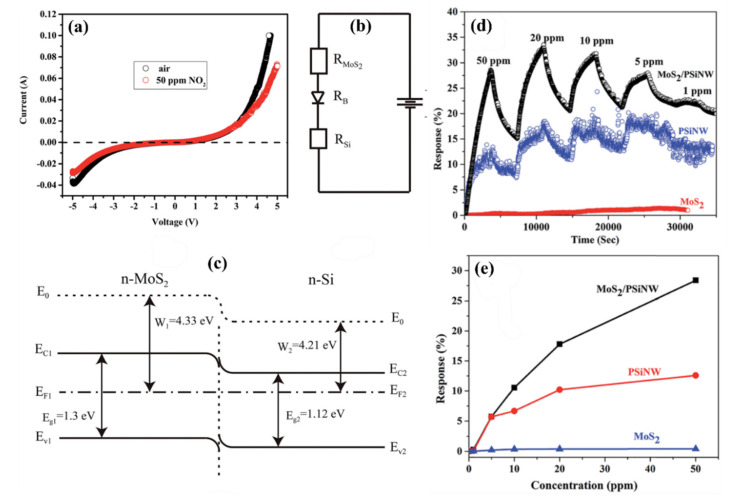
(**a**) I–V curves of MoS_2_/SiNW heterojunctions in air and NO_2_, (**b**) Equivalent electrical resistance model of MoS_2_/SiNW heterojunctions schematic illustration of using CVD to grow MoS_2_ nanosheets on PSiNWs, and (**c**) Schematic illustration of the energy band of MoS_2_/SiNW heterojunction structures, and (**d**) Dynamic response in different NO_2_ concentrations, and (**e**) response values of NO_2_ concentrations. Reproduced from [164].

Another approach for NO_2_ sensing was presented by S. Zhao et al. by using MnS_2_/SiNWs heterojunction [164]. In such sensor, the MoS_2_ nanosheets were deposited, using sulfurization of Mo film (pre-deposited using magnetron sputtering) directly on the top of n-type PSiNWs (obtained by Ag-MACE). In this study, Ag printed electrodes were deposited on the top of the structure to provide Ohmic contact. Since the work function of the n-MoS_2_ is higher than n-Si, then the Fermi level of Si is higher (it is schematically presented in the diagram in Figure 23a–c), electrons are transferred to the MoS_2_ towards the MoS_2_/Si interface. In this structure, NO_2_ is absorbed mostly on the sulfurs’ vacancies in the MoS_2_ structure. This reaction is a chemical sorption where NO_2_ is acting as an electron acceptor. Because of this, during the reaction with NO_2_, electrons from the conduction band are captured resulting in the resistance increment of the MoS_2_ and also increasing of the boundary barrier (depletion region) in the heterojunction, which leads to increase of the overall resistance of sensing structure. It needs to be emphasized that these experiments were carried out in an oxygen-free atmosphere using pure N_2_ as a carrier gas, where the influence of oxygen in the sensing mechanism was not considered. For this reason, it is difficult to say how the structure is working in the air conditions. However, it is shown that the MoS_2_/SiNWs heterojunction shows a significantly higher response to NO_2_ than MoS_2_, or SiNWs themselves (Figure 23d,e).

#### 5.3.3. Heterojunctions with Organic Semiconductors

In recent years the organic semiconductors–π-conjugated polymers like polyaniline, polypyrrole (PPy), polythiophene, their derivatives and many others are examined as candidates for new generation gas sensing materials. In many cases such polymers show high sensitivity, room temperature operation and selectivity [165]. However, the weakest sides of these materials are a lack of long-term stability and poor resistance to: oxidation, radiation, temperature, and some other chemical agents. Organic semiconductors are also often used to create heterojunctions in hybrids/composites with other materials which lead to their applications in photovoltaics, light sources, sensors, and general electronics.

**Figure 24:**
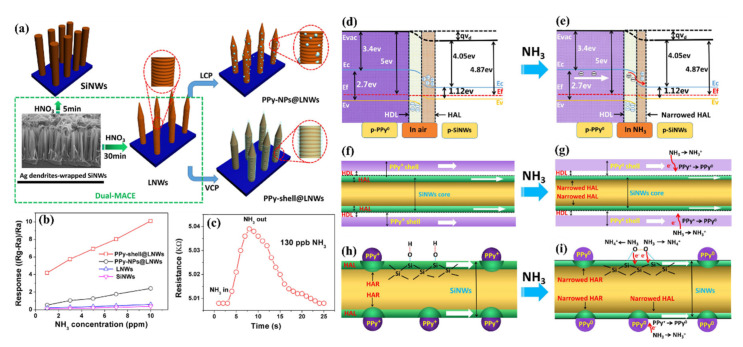
(**a**) Schematic illustration of the major processes involved in the fabrication of PPy-NPs@LNWs and PPy-shell@LNWs, (**b**) Response comparison of PPy-shell@LNWs, PPy-NPs@LNWs, bare LNWs and bare SiNWs, (**c**) Dynamic response of PPy-shell@LNWs to 130 ppb NH_3_ at RT. Schematic illustrations showing the NH_3_-sensing mechanism of PPy-shell@LNWs and PPy-NPs@LNWs: (**d**,**e**) Energy band diagrams of a PPy-SiNWs junction in air and in NH_3_; (**f**–**i**) conduction channel change before and after NH_3_ adsorption for PPy-shell@LNWs (**f**,**g**) and for PPy-NPs@LNWs (**h**,**i**). Reproduced from [166], with permission from Elsevier, 2020.

Qin et al. presented studies about SiNWs functionalized by PPy for NH_3_ [166] and NO_2_ [117] sensing. In first work, NH_3_ sensing was compared both PPy NPs decorate and PPy shell coated loose SiNWs (LNWs) array prepared using double step Ag-MACE method. The PPy-NPs and PPy-shells were applied on the SiNWs array using liquid chemical polymerization (LCP) and vapor chemical polymerization (VCP) processes, respectively (Figure 24a). Functionalization by PPy significantly improved the response to NH_3_ in comparison to SiNWs and LNWs. PPy-shell structures show few times higher response than PPy-NPs (Figure 24b,c). Since PPy is a p-type semiconductor and p-type SiNWs were used, then the structure is a p-p heterojunction. Because the work function of PPy is higher than Si, then in this heterojunction electrons flow from Si to PPy and at the interface HAL and HDL are created on Si and PPy side, respectively. During the redox interaction between NH_3_ and PPy electrons are donated to the PPy where they can easily diffuse to the HAL in the junction, as it is schematically are shown in the energy diagrams of the junction in the Figure 24d,e. Because of the HAL is shrinking, it causes an increase in the structure resistance. The study shows that the size of the heterojunction plays a crucial role here, since in the case of core-shell structures, PPy is practically covering whole surface of the SiNW while PPy NPs are covering the surface locally in some spots. Because of that, in shell structures, all the HAL is regulated by gas interaction while in the case of NPs only local hole accumulation regions are changed (Figure 24f–i). The PPy-SiNWs hybrid structures, especially shell ones, show promising NH_3_ sensing properties like high response, fast response time, and relatively high selectivity towards acetone, methanol, H_2_, ethanol, and CH_4_.

**Figure 25:**
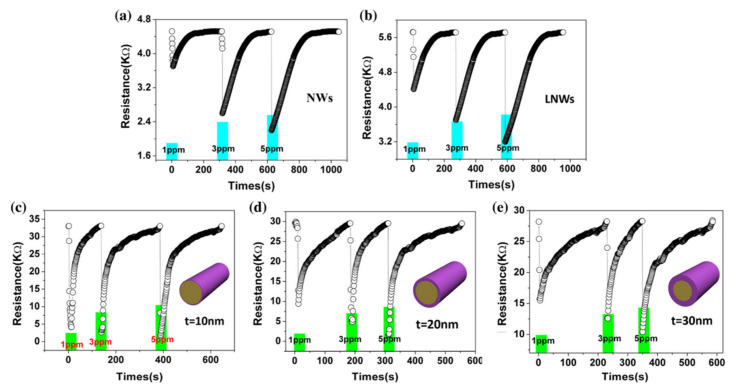
Dynamic response of the sensors based on NWs: (**a**) LNWs, (**b**) LNWs/PPy-10, (**c**) LNWs/PPy-20 (**d**), and LNWs/PPy-30, and (**e**) to varying concentration of NO_2_ at room temperature. Reproduced from [117], with permission from John Wiley and Sons, 2020.

In another work [117], the authors concentrated only on the PPy-shell@LNWs structures and their NO_2_ sensing properties at sub ppm and ppm concentration range. They focused here on the PPy-shell film thickness influence on the NO_2_ sensing. It was found out that the thinner the shell film is the higher the structure sensitivity is. This can be observed in Figure 25 where dynamic responses of bare SiNWs and LNWs, as well as PPy-shell@LNWs are presented with 10, 20, and 30 nm thick PPy-shells, respectively. In this article, authors put a lot of attention to the sensing mechanism of the structure. It was shown that the conductivity of this structure is mainly dependent on the PPy layer resistance and the HAL size in the PPy-Si interface (considered as a HAL resistance), while the influence of much higher SiNWs resistance is negligible. Therefore, they propose a parallel resistance model where only PPy and HAL resistances are considered. When the structure is exposed to NO_2_, electrons are extracted from the PPy, which increases its conductivity via increasing of holes concentration and simultaneously shallowing the HAL which is competitively increase the resistance of HAL. As the resistance of the structure is significantly decreasing during the NO_2_ exposure, the overall structure resistance model may be simplified by focusing on the carrier transport via the PPy shell. Thanks to this effect they explain that the very thin PPy shell have much lower initial hole density (high initial resistance in the air) because of the electron injection from Si during the hetero-contact creation. The NO_2_ adsorption causes then the much higher relative increase of the free holes concentration (higher resistance response) in thin PPy shell than in thicker one. It has to be stressed that authors obtained here very high and rapid response to NO_2_ at low concentration level and presented the limit of detection of 50 ppb at RT and RH = 30%. They also presented the selectivity to the same gases as in the case of the NH_3_ sensor described above. However, the authors stressed here that, at higher RH (higher than 50%), the sensing properties are dropping down.

### 5.4. Carbon Materials

**Figure 26:**
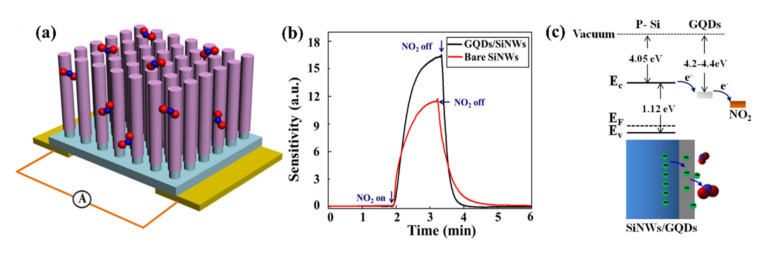
(**a**) Schematic diagram of the GQDs/SiNW array-based gas sensor. (**b**) Sensitivity responses of the SiNWs array with and without decoration of GQDs to NO_2_ (500 ppm) at room temperature. (**c**) Energy band diagram of the GQDs/SiNW heterojunction. Reproduced from [167], with permission from IOP Publishing, 2020.

Graphene quantum dots (GQDs) have various applications in biological imaging, photovoltaics, composites, and sensors due to their unique atomic arrangement. Li et al. proposed a novel structure based on a GQD modified SiNW array for sensitive detection of NO_2_ [167]. The scheme of the device is shown in Figure 26a. As shown in Figure 26b, in comparison with the bare SiNW array, the resistor based GQDs/SiNW array sensor demonstrates higher sensitivity, quicker recovery, higher stability and reproducibility. Real-time detection shows that a trace amount of NO_2_ with a concentration as low as 10 ppm could be efficiently identified at room temperature [167].

Figure 26c reveals the corresponding energy band diagram of the GQDs/SiNW heterojunction [167]. Due to the differences between the conduction band position of Si and the work function of the GQDs, the conduction band of Si would be bent, thus leaving an adequate built-in electric field to appear at the GQDs/SiNW interfaces. In this way, electrons could be easily extracted from SiNWs and even stored in the GQDs layer [167]. When the GQDs/SiNW array-based detectors are exposed to NO_2_ atmosphere, electrons would be easily transferred to the absorbed NO_2_ molecules due to their high electron-withdrawing ability and the rich electron storage in the GQDs layer. The electron loss from the SiNWs would induce the holes accumulation and electrical conductivity enhancement of p-type SiNWs, which result in a rise of the current flowing through the sensing structure. What is more, the quicker the electron transfer, the lower the response time would be [167]. When NO_2_ is removed from the test chamber, the electron captured by NO_2_ will be returned to the GQDs/SiNW array and the hole accumulated in the SiNWs would be consumed rapidly due to the high electron transportation ability of the GQDs layer, thus a short recovery time during detection could be obtained. Therefore, GQDs could not only protect the SiNW array from oxidation but also improve the electron transfer between the sensing structure and the analytes, which benefits both the response and recovery processes during detection [167].

**Figure 27:**
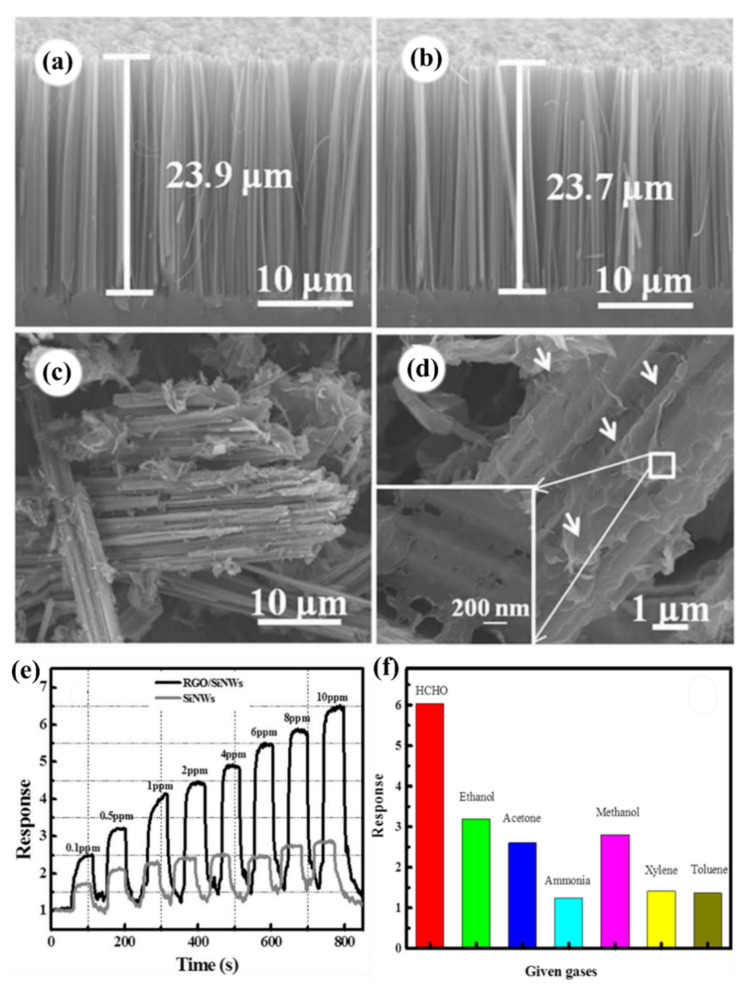
Cross-sectional SEM images of (**a**) n-SiNWs. (**b**) p-SiNWs. (**c**) SEM image of RGO@n-SiNWs with HF treatment, (**d**) Zoomed-in SEM image of RGO@n-SiNWs with HF treatment, (**e**) dynamic response of n-SiNWs and RGO@n-SiNWs from 0.1 to 10 ppm HCHO, (**f**) The response of n-SiNWs and RGO@n-SiNWs for seven types of common VOCs (10 ppm) at 300 °C. Reproduced from [115].

Song et al. prepared SiNWs with high specific surface area via MACE, and then are wrapped by reduced graphene oxide (RGO) to form a p-n junction for low concentration detection of formaldehyde at 300 °C [115]. The SEM images of the bare and RGO@n-SiNWs are shown in Figure 27a–d. After wrapping RGO, the specific surface area increases two-fold demonstrated by N_2_ absorption-desorption isotherm. More importantly, due to the formed p-n junction, the RGO@n-SiNWs reveals a good sensitivity and selectivity with interfering gases presented in Figure 27e,f. The sensing mechanism is the same as what we have discussed previously for GQD modified SiNWs. Although in this case the sensor is exposed to the reducing agent, the functionality of GQDs and RGO are almost the same [115].

**Figure 28:**
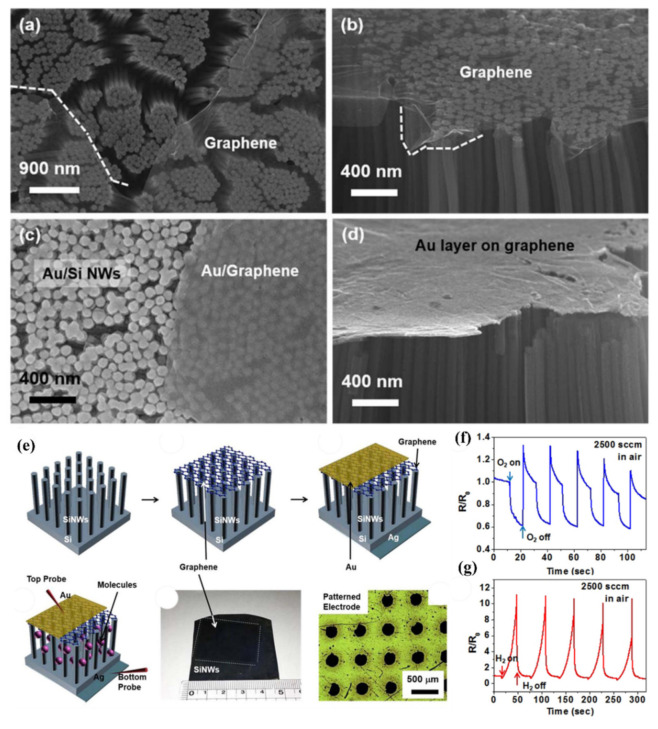
Plan-view image of the graphene/Si-NW heterostructure where, (**a**) dark graphene sheet on tips of SiNWs, (**b**) tilted-view image of graphene/Si NWs confirming the uniform contact between graphene and Si NWs, and (**c**) plan-view image of an Au film on graphene and tips of Si NWs. A continuous Au film is formed only on graphene where (**d**) tilted-view image of Au/graphene/Si NWs with a continuous Au film is well placed on the graphene/Si NWs, (**e**) fabrication process of the device. Normalized resistance responses of graphene/SiNW heterostructure molecular sensor under repeated exposures of (**f**) O_2_ and (**g**) H_2_ gases in air at room temperature. Exposure intervals of O_2_ and H_2_ gases are 10 s and 30 s, respectively. Reproduced from [168].

There is also a study where graphene plays a key role in preventing tips of vertical SiNWs from being bundled, thereby making SiNWs stand on Si wafer separately from each other under graphene, a critical structural feature for the uniform Schottky-type junction between Si NWs and graphene [168]. The SEM images and fabrication process are illustrated in Figure 28a–e. This structure showed sensitivity towards O_2_ and H_2_ gases, as depicted in Figure 28f,g [168]. The mechanism is almost the same as what we discussed before.

### 5.5. Chemical Surface Modification

Beside the decoration of metal NPs and heterojunctions with semiconducting materials also chemical surface modification of SiNWs was reported in the literature which is believed to be continued as one of efficient functionalization ways in the future.

**Figure 29:**
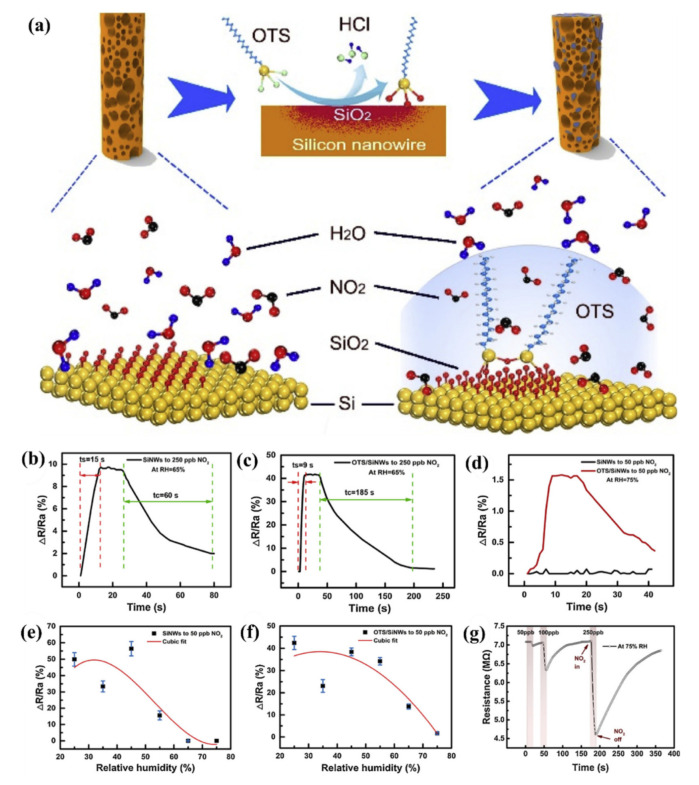
(**a**) Schematic illustration of construction of OTS-modified porous SiNWs for NO_2_ detection under high humidity condition, (**b**) Dynamic responses to 250 ppb NO_2_ at 65% RH for SiNWs sensor and (**c**) OTS/SiNWs sensor, (**d**) dynamic responses of OTS/SiNWs and SiNWs sensors to 50 ppb NO_2_ at 75% RH. (**e**) response value versus relative humidity and corresponding cubic fits (red line) for SiNWs sensor, (**f**) OTS/SiNWs sensor towards 50 ppb NO_2_, and (**g**) dynamic response of OTS/SiNWs sensor to varying concentration of NO_2_ at 75% RH. Reproduced from [116], with permission from Elsevier, 2020.

For example, Qin et al. reported the functionalization of SiNWs by octadecyltrichlorosilane (OTS) to enhance the humidity resistance during NO_2_ sensing [116]. In this case, the SiNWs arrays prepared by MACE were conjugated by OTS from 1 vol% toluene suspension at RT. Thanks to that hydrophobic monolayer of OTC was formed on the SiNWs surface. Such a monolayer, thanks to hydrophobic properties, is shielding the surface of the SiNWs from the H_2_O molecules, but is transparent to NO_2_, as is schematically shown in Figure 29a. Authors showed clearly that the addition of OTS improve the sensing properties of the SiNWs at higher RH levels (Figure 29b–g). Sensor shows reasonable responses even to tens of ppb of NO_2_ at RH reaching level of 75% which is a huge improvement in comparison to bare SiNWs. What is more, sensor response time is even higher when OTS functionalization is used while regeneration time is slightly worse but is still reasonable. Presented limit of the detection of that structure is of 5 ppb and 50 ppb for RH 55% and 75%, respectively.

Haick et al. presented several reports concerning molecularly modified SiNWs based gas sensors for selective VOCs sensing [28,169,170,171]. In these reports SiNWs FETs are modified by different chemical agents like propyl, propynyl, chloro(phenyl)silanes, different chlorides, and acids. Mutual, differently modified SiNWs sensors are examined there for reactions to many different polar and nonpolar VOCs. Then responses are compared using different method and calcifications including neural networks for different applications like recognition of multicomponent gas mixtures and breath analysis. These works clearly shows that molecular surface modification of SiNWs may have huge influence on their VOCs sensing behavior where current, voltage, or carrier mobility can be taken as sensing signals.

Gao et al. presented the surface modification of SiNWs by (3-Aminopropyl) triethoxysilane for odorant binding proteins like nonanoic acid vapor detection for biosensing application [172]. In another work from this group, Liu et al. reported (3-Aminopropyl) dimethylethoxysilane monolayer on SiNWs array for detection of trinitrotoluene vapors [173]. In both cases, the authors showed that proposed surface modification of the SiNWs is improving or even causing sensing properties of the structure for specific analytes.

### 5.6. Integration of Multiple Functionalization Methods

**Figure 30:**
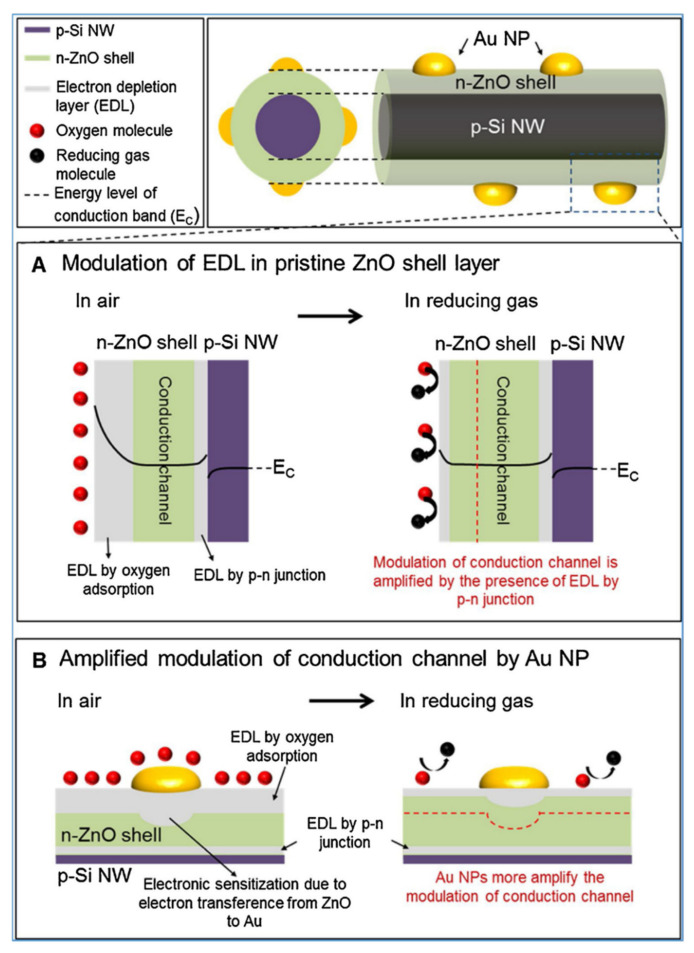
Sensing mechanism in the Au-functionalized SiNWs/ZnO core-shell gas sensor, (**a**) Formation of the depletion layers in the pristine SiNWs/ZnO core-shell layer and (**b**) Expansion of the electron depletion layer in the presence of Au. Reproduced from [174], with permission from Elsevier, 2020.

**Figure 31:**
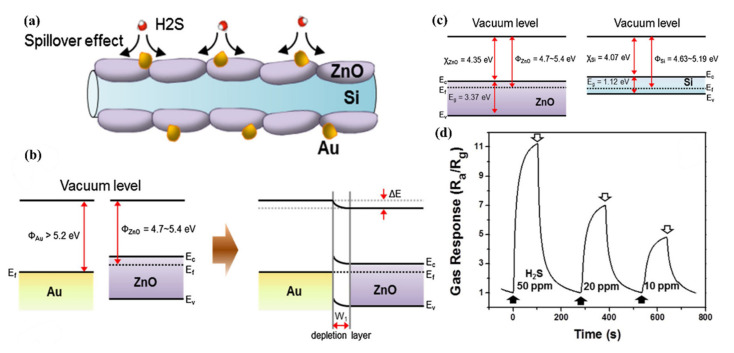
(**a**) Schematic outline showing spillover effects due to the presence of Au NPs, (**b**) energy band structure of ZnO/Au upon the formation of heterointerfaces, and (**c**) energy band structure of ZnO/p-Si prior to contact, (**d**) Gas response for different concentrations of H_2_S gas at 300 °C. Reproduced from [174], with permission from Elsevier, 2020.

There are several reports which combines different functionalization methods to improve the performance of the SiNWs sensors. Choi et al. presented SiNWs with Zn shells functionalized by AuNP for H_2_S sensing [174]. The SiNWs/ZnO core-shells were synthesized by MACE and the thermal evaporation of Zn is promoted with Au NPs generated on the surface for H_2_S sensing [174]. The small bonding energy of H-HS, small size of H_2_S molecules, catalytic role of Au, intrinsically high sensitivity of ZnO, and formation of Si/ZnO and Au/ZnO heterojunctions are the primary causes for the good response to H_2_S gas. Authors proved that the sensing behavior is governed by ZnO shell with n-type semiconducting behavior and they have calculated the Debye length for ZnO at elevated working temperature (300 °C). According to the authors since ZnO shell thickness is larger than the Debye length of ZnO, the electrical conduction will be mainly limited to the ZnO shell layer rather than the Si core. Apart from obvious reaction between H_2_S and oxygen species on the surface of ZnO that modulates the width of depletion layer at the surface of ZnO, in the SiNWs-ZnO core-shell, additional factors, namely: (i) heterojunctions between ZnO/Au, (ii) the catalytic effects of Au, and (iii) ZnO/Si heterojunction must be considered. Figure 30 schematically shows the associated sensing mechanism. Regarding Au NPs, due to its catalytic effect, gas molecules are dissociated, in a so-called spill-over effect, and transferred onto the surfaces of the ZnO shell. In fact, Au acts as a very effective adsorption site to adsorb H_2_S gas (Figure 31a). Because of the difference in work function of ZnO and Au, electrons are transferred from ZnO to Au which leads to up-come of a depletion layer at the interface (Figure 31b). After exposure to H_2_S gas, the released electrons return to the surface of ZnO and the width of the depletion layer decreases significantly. This modulation of the resistance causes an increase of the response to H_2_S gas. Through the adsorption of H_2_S gas, the resistance will decrease. It is supposed that the amount of decrease in resistance is similar for both cases, the sensor response of ZnO in the presence of the ZnO/Au interface will be larger than that of ZnO without ZnO/Au interfaces. Accordingly, the modulation of the resistance by the ZnO/Au heterojunctions will contribute to improve the H_2_S sensing performance. Therefore, in the case of Au, both effects will enhance the response of the sensor [174].

Regarding to the junction of ZnO and p-SiNWs, their work functions should be considered. The work function of ZnO is in the range of 4.7–5.4 eV while the work function of p-Si is 4.63–5.19 eV. The energy band diagram of ZnO and p-Si is depicted in Figure 31c. Therefore, it is more likely that the work function of ZnO is higher than that of p-Si. However, there is also the possibility that the work function of p-Si is higher than that of ZnO. According to the authors [174], the only case that contributes to the sensor response is the one that work function of p-Si is higher than that of ZnO. Similar to what they have mentioned to justify the improvement of the sensor response in the junction of ZnO/Au, authors [174] used the same idea to justify the improvements in ZnO/p-Si junction. They also investigated the selectivity mechanism for H_2_S gas towards some interfering gases. The response of this sensor is presented in Figure 31d for 50, 20 and 10 ppm of H_2_S gas operating at 300 °C. However, they still suffer from high energy consumption because of their operation at high temperature rather than RT.

**Figure 32:**
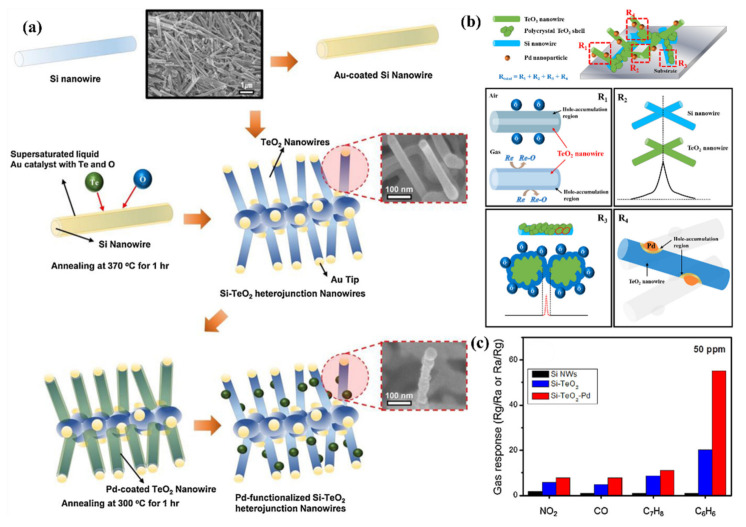
(**a**) Fabrication processes of the Pt-functionalized TeO_2_-branched Si nanowires, (**b**) schematic diagram showing the four mechanisms being operated in the Pd-functionalized branched nanowires: (R_1_) modulation in the hole accumulation along the branch TeO_2_ nanowires, (R_2_) modulation of the potential barrier at the networked homojunctions between the branch TeO_2_ nanowires, (R_3_) modulation of the potential barrier at the boundaries of the nanograins and (R_4_) modulation of the potential barrier at the Pd-TeO_2_ heterojunctions (including additional Pd effects), and (**c**) column bar graph showing the variation of sensor responses by addition of TeO_2_ branches, Pd-functionalization, and varying the gas species at 50 ppm. The sensing temperature was 200 °C. Reproduced from [175], with permission from Elsevier, 2020.

In another work from this group [175], in order to increasingly improve the benzene (C_6_H_6_) gas sensing performance of SiNWs, they fabricated TeO_2_-branched SiNWs that were subsequently decorated with Pd nanoparticles. Figure 32a shows the fabrication process of the NWs. Compared to other gases, they demonstrated superior sensor responses of both the pristine and Pd-functionalized branched NWs to C_6_H_6_ gas operating at 200 °C. By means of Pd functionalization, the sensor response to C_6_H_6_ gas is significantly enhanced while the response and recovery times are significantly reduced. According to Figure 32c, the sensor responses of the as-prepared and Pd-functionalized branched NWs exhibited superior sensor responses to C_6_H_6_ gas, 20.18 and 55.19, respectively. In the following sections several sensing mechanisms will be briefly investigated. Before that, it is necessary to mention that both Si and TeO_2_ showed p-type behaviors and according to authors since the TeO_2_ branched structures nearly completely cover the SiNWs, the main hole current will flow through p-TeO_2_ rather than p-Si. The change in the resistance will be along the length direction of each TeO_2_ branch, which is denoted as R_1_ in Figure 32b. The networked structures of TeO_2_ branches generate numerous homojunctions. The built-in potentials at the junctions of the networked branches or NWs will be altered by the adsorption and desorption of gas molecules (R_2_). These homojunctions will generate potential barriers, bringing about a modulation of the resistance. The potential barriers of homojunctions made by grain-like structures of TeO_2_ on the stem NWs in addition to the TeO_2_ branches will be changed by the adsorption and desorption of gas molecules (R_3_). The modulations of the potential barrier at the Si-TeO_2_ heterojunctions and the last one is catalytic effect of Pd NPs (R_4_).

**Figure 33:**
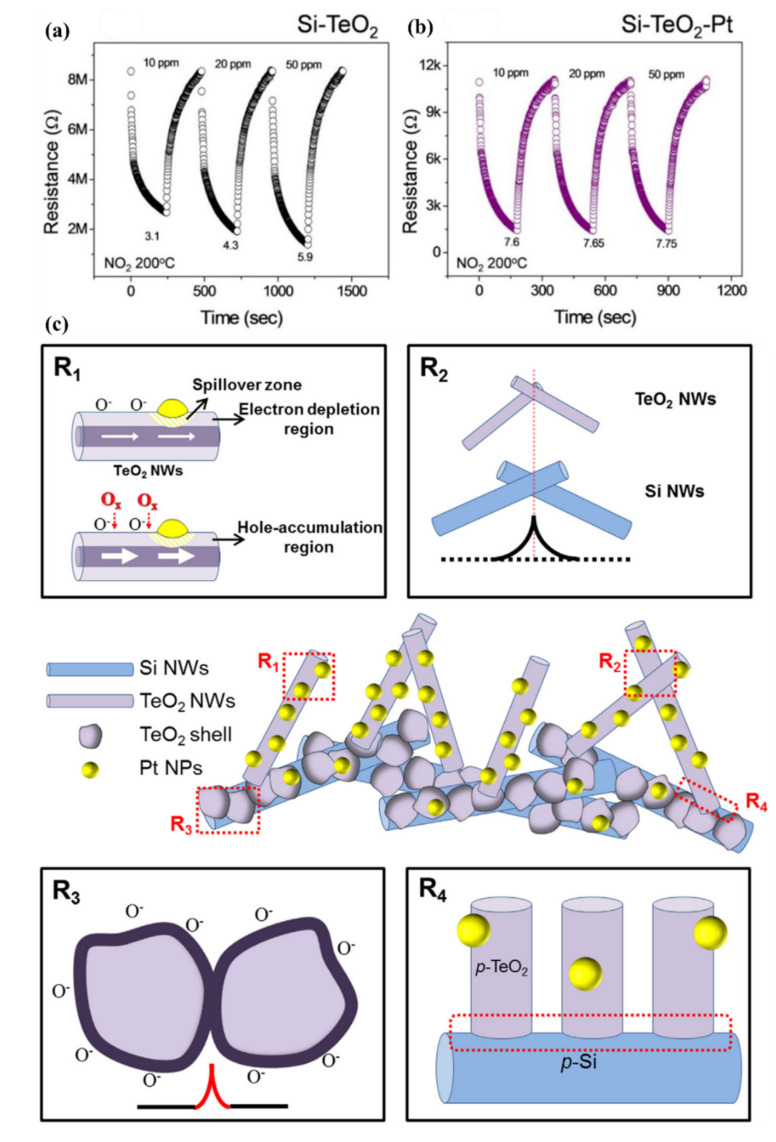
Dynamic resistance curves of (**a**) pristine and (**b**) Pt-functionalized branched nanowires at NO_2_ concentrations of 10, 20, and 50 ppm. (**c**) Schematic diagram showing the four resistance mechanisms operating in Pd-functionalized branched nanowires: (R_1_) modulation of depletion width along the branched TeO_2_ nanowires (including additional Pt effects), (R_2_) modulation of the potential barrier at networked homojunctions between branched TeO_2_ nanowires, (R_3_) modulation of the potential barrier at boundaries of the nanograins, and (R_4_) modulation of the potential barrier at Si-TeO_2_ heterojunctions. Reproduced from [176], with permission from Elsevier, 2020.

In a report from Bang et al., a toluene (C_7_H_8_) sensor using SiNW-TeO_2_ heterostructure sensitized with Pt NPs was demonstrated [176]. Ag-MACE process was employed to fabricate SiNWs and then dense TeO_2_ branches formed on the NWs followed by uniformly sputtering of Pt layers onto the surface. This structure could change the isolated PtNPs after a thermal treatment. They have investigated the effect of PtNPs on the sensing performance (Figure 33a,b) and revealed that the response of the SiNW-TeO_2_/Pt composite was four-fold higher than the Pt-absent sensor at the concentration of 50 ppm at 200 °C (optimal working temperature). As illustrated in Figure 33c, modulations of four resistances contribute to the sensing behavior. The enhancement of response due to Pt is attributed to several reasons e.g., the spillover effect (R_1_); active reaction site generation in interfaces of SiNWs, TeO_2_, and PtNPs; modulation of the potential barriers formed at the heterojunction interfaces between SiNWs and TeO_2_ as well as TeO_2_ and PtNPs (R_2_, R_4_). Furthermore, more detailed investigations showed the other cause behind the enhancement of response is homojunction interfaces of SiNWs and TiO_2_ NWs (R_2_, R_3_), and enhancement of chemisorption and dissociation effect of C_7_H_8_ [176]. Although this sensor shows good sensitivity with proper selectivity and repeatability, it still suffers from operating at high temperature as 200 °C which results in more energy consumption.

**Figure 34:**
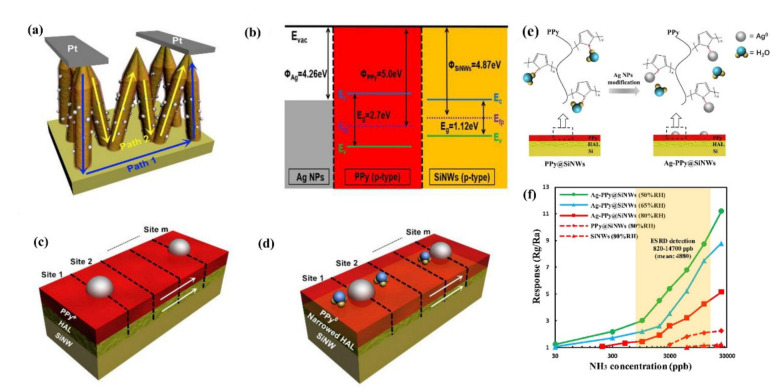
Schematic illustrations showing the NH_3_-sensing mechanism of Ag-PPy@SiNWs: (**a**) electrical conducting paths, (**b**) energy band diagram of an Ag-PPy@SiNWs junction in air. Conduction channel changes (**c**) before and (**d**) after NH_3_ adsorption for Ag-PPy@SiNWs. (**e**) Schematic diagram of anti-humidity effect induced by Ag NPs. (**f**) Response comparison of Ag-PPy@SiNWs, PPy@SiNWs and SiNWs at room temperature. Reproduced from [177], with permission from Elsevier, 2020.

In another paper of Qin et al., the dual-modulated composite array of Ag NPs-modified PPy@SiNWs core-shell structures (Ag-PPy@SiNWs) are developed for sensitive response to NH_3_ at high ambient humidity for biomedical applications [177]. The schematic of the device is presented in Figure 34a. The sensor’s response usually drops down at high ambient humidity. As mentioned before many efforts failed to achieve a robust sensor working in this situation. Y. Qin et al. fabricated a loose array of pristine SiNWs by a repeated MACE. Then Ag-PPy@SiNWs array prepared in one-step via vapor phase polymerization (VPP) of Py on SiNWs with AgNO_3_ as oxidant. PPy hetero-shell and Ag NPs contribute to NH_3_-sensing performance under high ambient humidity. Ag-PPy@SiNWs can detect NH_3_ as low as 200 ppb at room temperature and 80% RH. In the case of PPy@SiNWs, the ultrathin PPy coating layer modulates the conductivity of PPy@SiNWs by creating PPy-SiNWs heterojunction which significantly enhanced the response to NH_3_ compared to bare SiNWs. However, Ag NPs could further improve NH_3_-sensing characteristics by modulation of the conductivity. Figure 34b shows the energy band diagram near the Ag-PPy-SiNWs heterojunction before the Fermi levels equilibrium. Due to the difference in work functions, electrons are transferred from p-Si to the PPy and make HAL at the Si interface. Because of the different conductance mechanism for organic PPy which is π-π conjugation the charge carriers can transfer throughout the whole network. Thus, the whole this conductive shell and the created HAL dominate the axial conduction channels in the PPy/SiNWs. NH_3_ adsorption on the Ag-PPy@SiNWs causes the number of free holes to decrease due to the co-contribution of electronic sensitization (ES) and catalytic sensitization (CS) introduced by attached Ag NPs. As for CS, the tiny Ag NPs attached on the shell surface are highly active and the catalytic activity of Ag NPs facilitates much more NH_3_ molecules to be adsorbed via so-called “spillover” and “back spillover” effect, giving rise to a considerable decrease in holes concentration in PPy shell as well as an increase in active sites. In terms of ES, NH_3_ adsorption on the tiny Ag NPs can decrease the work function of Ag NPs effectively. It further induces an enhanced donation of electron from the attached Ag NPs to PPy shell during NH_3_ sensing. Figure 34c,d shows the conduction channel change before and after NH_3_ adsorption. Authors also proved that the Ag NPs on the PPy weaken the humidity interference. It is noteworthy that the tertiary nitrogen groups are the general coordination sites of water molecules adsorbed on the polymer surface. Thus, during PPy polymerization, the as-formed Ag prefers to coordinate with –N groups in PPy, which inhibits the further adsorption of water on the same –N groups. Meanwhile, the attached Ag NPs is highly hydrophobic. As shown in Figure 34e, Ag NPs having strong hydrophobicity on the PPy chain make almost no water adsorption on the PPy shell. The less water molecule adsorption further promotes the CS and ES effects. Figure 34f shows brief comparison for the response of Ag-PPy@SiNWs, PPy@SiNWs and SiNWs at room temperature [177].

**Figure 35:**
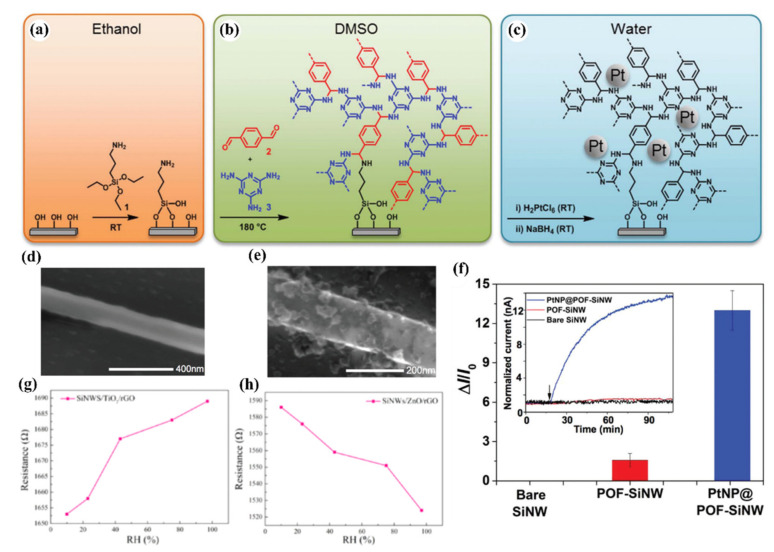
Schematic representation of (**a**) 3-aminopropyltrimethoxysilane grafting to the silanol-terminated surface of a SiNW, (**b**) the covalent modification process of the SiNW surface with melamine-terephthaldehyde-based POFs, (**c**) the metalation of a POF-SiNW via an in situ chemical reduction. Molecules 1–3: APTES, melamine, terephthaldehyde, respectively. SEM images of (**d**) bare SiNW, and (**e**) SiNW decorated with POFs, (**f**) normalized response upon exposure to 3000 ppm of methanol vapor for bare SiNW, POF-SiNW and PtNP@POF-SiNW sensors, and both (**g**) and (**h**) show the resistance of the ZnONPs-SiNW/rGO and TiO_2_NPs-SiNW/rGO as a function of humidity. Reproduced from [15]; and Reproduced from [178], with permission from RSC Pub, 2020.

A. Cao et al. demonstrated the functionalization of SiNW surfaces with pours organic frameworks (POFs) and explored its effect on the electrical sensing properties of SiNW-based devices for detection of methanol vapor [178]. The authors have done the modification by polycondensation of melamine and terephthaldehyde on the amine-modified SiNWs as depicted in Figure 35a,b. Pt NPs were formed in these POFs by impregnation with chloroplatinic acid followed by a chemical reduction using sodium borohydride (NaBH_4_), as illustrated in Figure 35c. SEM images of bare SiNW, and SiNW decorated with POFs are presented in Figure 35d,e. They have investigated the humidity effect on the POFs-modified SiNWs structure and have seen that the signal increased by **~**2 orders of magnitude. This is attributed to the presence of the flaky POF structure because of high specific surface area and its richness in N atoms. The high specific surface area as we have discussed before will contribute to a fast and increased uptake of water into the porous structure, resulting in a pre-concentration effect. The sensor used as a transistor and brought into depletion mode by applying special constant voltage to back-gate and source-drain and meanwhile monitoring the current passes the channel [178]. As shown in Figure 35f for the PtNP@POF-SiNWs authors observed enhanced response compared to the bare SiNWs and POF-SiNWs towards methanol. The main reason behind that related to an enhanced dissociation of the surface silanol groups of the SiNWs. Also, authors stated that the capacitive coupling of the gate potential via these PtNPs can contribute to the response. This effect previously reported in [142]. They have also investigated the selectivity of the sensor toward some other VOCs like ethanol, isopropanol, and acetaldehyde vapors. They justify the selectivity trough the parameters related to dielectric constants of the gases. The higher the dielectric constant, the higher the polarity, and the more favorable to the dissociation.

There is a report in 2020 concerning the respiratory monitoring using SiNW/reduced graphene oxide (rGO) decorated with ZnO and TiO_2_ NPs [15]. In this structure, the SiNWs are fabricated by MACE and then the rGO placed on the top of NWs to form the top electrode. The Schottky-structure-based sensor in this work can be regulated by TiO_2_ and ZnO NPs. Meanwhile in this sensor, the Fermi level of graphene can be regulated by water molecules which is believed to have p-type doping effect on rGO [15]. Upon exposure to humidity, the reducing effect of the water molecules helps the electrons to transfer to the ZnO conduction band leading to an increase in the carrier concentration and the conductivity of the nanostructure as illustrated in Figure 35g. On the other hand, TiO_2_ increases the oxygen vacancies on the sensor surface and helps to dissociate water molecules, making the resistance value increase with increasing humidity. The results are presented in Figure 35h. The response times of ZnONPs-SiNW/rGO and TiO_2_NPs-SiNW/rGO were 49 s and 67 s, and the recovery times were 24 s and 43 s, respectively for 43% RH (the base line is 23% RH); the response and recovery times of ZnONPs-SiNW/rGO were better than those of TiO_2_NPs-SiNW/rGO [15].

Table 5 provides a complete overview of all papers using organic or inorganic materials (sometimes combined with NPs) as a functionalization method for SiNWs gas sensors.

## 6. Conclusions

In this article, the development of SiNW gas sensors in recent years has been reviewed. The content begins to describe the NW fabrication for both the bottom-up and top-down approaches. Later, the evolution of lithography technique for patterning nano-scale features was briefly introduced and then further discussions included the other techniques e.g., E-beam and sidewall transfer lithography. The etching methods to create NWs and how to form the ohmic contacts with low contact resistance to the NWs were also presented. Afterwards, the main theory of gas sensing of NWs was described. At first, the basic definitions of oxidizing and reducing agents were explained and then the content was extended to present the functionalization methods, doped junctions with different semiconducting materials and chemical surface sensitization. The resistor configuration, which is the most common device for gas sensing, was compared to field effect transistor one. Furthermore, the challenges, difficulties and the advantages/disadvantages of the applied technologies and methods were also discussed. In this part, the influence of morphology and size effect on gas sensing of the pristine or suspended NWs with an efficient self-heating utilization was described. The content highlighted how the sensitivity could be tailored or improved by using heterojunctions with: MOXs; conducting polymers or with organic and inorganic semiconductors; graphene and its derivatives, and also combination of these heterojunctions with metal nano particle decoration. The physical explanation how each material could functionalize the NWs and change the gas sensing properties was provided.

This work provides valuable knowledge which may be utilized in the future to design and evaluate or tailor the active part of the sensors for specific analyte sensing.

## 7. Future Prospective

More than Moore approach provides novel applications where gas sensors could be integrated with other advanced devices. Such designs improve the functionality of the sensors with high efficiency.

The recent development of SiNW devices enables the sensitive and rapid analysis of gas species. It is believed that these advanced sensing devices would be commercially developed and could be widely used in our daily life. Their novel applications include environmental testing for air and water quality as well as human safety like uncovering and diagnosing disease and also food safety. For example, with the rapid growth of the nanotechnology, the SiNW gas sensors can become a promising candidate to monitor the air quality simply by using portable devices. For such a case, the SiNW gas sensor chip can be transferred on the readout chip and the signals could be amplified and analyzed for various applications. Furthermore, the arrays of gas sensors in the single chip can be functionalized for the gas sensing selectivity and such a response pattern across the arrays can be then analyzed by a software analysis program for pattern recognition.

Although many technological options are now available, more work has to be performed to obtain solutions for a better selectivity and faster response for a large number of gas molecules in the near future.

## Figures and Tables

**Table 1:** Several typical phases in the contact formation of silicides. Reproduced or adapted from [87], with permission from Springer Nature, 2020.

Phase	Reaction Temperature (°C)	Crystal Structure	Shottky Barrier Height (eV)	Interfacial Plane Structure	Ref.
MnSi	650	Cubic	0.65	MnSi (−2 −1 4)‖Si (3 4 5)	[88]
				MnSi [1 −2 0]‖Si [3 −1 −1]	
CoSi_2_	800	Cubic	0.64	CoSi_2_ (−1 1 1)‖Si (−1 1 1)	[89]
				CoSi_2_ [1 1 0]‖Si [1 1 0]	
PtSi	520	Orthorhombic	0.88	PtSi (1 0 1)‖Si (1 1 1)	[90]
				PtSi [0 1 0]‖Si [1 −1 0]	
NiSi	450	Orthorhombic	0.65	NiSi (−1 1 0)‖Si (1 −1 1)	[91]
				NiSi [0 0 1]‖Si [1 1 0]	
NiSi_2_	300~650	Cubic	0.66	NiSi_2_ (1 1 1)‖Si (1 1 1)	[92]
				NiSi_2_ [−1 1 0]‖Si [−1 1 0]	

**Table 2:** Overview of Electrostatically Formed Nanowires gas sensors for the detection of different gases.

Year	Approach	SiNW Size	Functionalization	WT	Target(s)	Detection Limit	Ref.
2018	TD	D: 29–56 nm	Bare	RT	VOC	No Data	[126]
2017	TD	D: 20 nm	Bare	RT	Ethanol	26 ppm	[123]
2017	TD	No Data	Bare	50–60 °C	VOC	50 ppm	[127]
2016	TD	D: 29 nm	Bare	RT	Ethanol, Acetone	∼26 ppm ethanol,∼40 ppm acetone	[128]
2015	TD	D: 16–46 nm	Bare	RT	Ethanol	100 ppm	[129]
2015	TD	D: 22–115 nm	Bare	RT	Ethanol	No Data	[130]

WT: working temperature, D, L and W stand for diameter, length and width of nanowires.

**Table 3:** Overview of bare SiNW gas sensors for detecting various gases.

	Year	Approach	SiNW Size	Functionalization	WT	Target(s)	Detection Limit	Ref.
Resistor	2018	TD	D: 30 nm	Bare	100 °C	H_2_	10 ppm	[112]
	2018	TD	D: 50 nm L: 10 μm	Bare	RT	NO_2_	10 ppm	[110]
	2018	TD	D: 50–125 nm L: 31 μm	Bare	RT	NO_2_	0.25 ppm	[111]
	2016	TD	D: 50–200 nm L: 25–30 μm	Bare	RT	NO_2_	18 ppb	[137]
	2016	TD	D: 90 nm L: 42 μm	Bare	RT	H_2_	50 ppm	[132]
	2016	TD	D: 100 nm L: 11–25 μm	Porous SiNWs	RT	NO_2_	50 ppb	[131]
	2016	TD	D: 550 nm	Bare	RT	NO_2_	1 ppm	[134]
	2016	TD	D: 90 nm L: 36 μm	Bare	RT	NO_2_	50 ppb	[133]
	2014	TD	W: 100 nm, L: 7.26 μm	Polycrystalline SiNWs	RT	NH_3_	2 ppm	[135]
	2014	BU	D: 150 nm L: ~20 μm	phosphorous in-situ doped	RT	NH_3_	2 ppm	[136]
FET	2018	TD	W: 100 nm	Bare	RT	NO_2_	1 ppm	[138]
	2017	TD	No Data	Bare	RT	Ethanol	No Data	[113]

WT: working temperature, D, L and W stand for diameter, length and width of nanowires.

**Table 4:** Overview of recent research works related to SiNW gas sensors functionalized by metal nanoparticles.

	Year	Approach	SiNW Size	Functionalization	WT	Target(s)	Detection Limit	Ref.
Resistor	2019	TD	W: 160 nm, L: 500 nm	Pd	RT	H_2_	0.01%	[141]
	2018	TD	D: 200 nm L: 30 μm	Pd-coated	RT	H_2_	2 ppm	[139]
	2018	TD	L: 20 μm	Ag	RT	NO_2_	10 ppb	[151]
	2017	TD	W: 215 nm	Ni-Silicide	250 °C	O_2_	No Data	[152]
	2017	TD	L: 30 μm	Ag	RT	NH_3_	330 ppb	[150]
	2017	TD	W: 160 nm	Pd	40 °C, 60 °C	H_2_	No Data	[143]
	2016	TD	D: 100–200 nm L: 8–12 μm	Pt/Pd	75 °C	H_2_	1 ppm	[147]
	2016	TD	D: 40–80 nm L: 22 μm	Pd	RT	H_2_	300 ppm	[145]
	2015	TD	D: 20–100 nm L: 13 μm	Pt, Pd, Ag, Au	RT	H_2_	15 ppm	[146]
	2015	TD	L: 1.35 μm	Pt, Au	RT	CO_2_	0.5 mbar	[149]
	2015	TD	L: 1 μm, W: 110 nm, H: 40 nm	Pd	RT	H_2_	0.1%	[140]
	2015	BU	D: 60 nm L: 1–4 μm	Ni	RT	Chlorine	5 ppm	[153]
FET	2020	TD	W: 70 nm L: 10 µm	Au	RT	NH_3_	1 ppm	[148]
	2015	TD	W: 70 nm, L:10 μm, H: 80 nm	Pd	RT	H_2_	0.01%	[142]
	2014	TD	W: 100 nm, L:1 μm, H: 50 nm	Pd	RT	H_2_	0.1%	[144]

WT: working temperature, D, L and W stand for diameter, length and width of nanowires.

**Table 5:** Overview of different junctions created with semiconductor materials with chemical surface sensitization as a modification method for SiNW gas sensors.

	Year	Approach	SiNW Size	Functionalization	WT	Target(s)	Detection Limit	Gases Checked for Selectivity	Ref.
Resistor	2020	TD	L: 1–3 μm,	TiO_2_ + Pt	200 °C	Toluene	10 ppm	CO, C_6_H_6_, NO_2_	[176]
	2020	TD	D: 100–200 nm, L: 8 μm	PdSe_2_	RT	Humidity	11%	-	[179]
	2020	TD	L: 20 μm	PPy + Ag	RT	NH_3_	200 ppb	Acetone, Methanol, Ethanol, Isopropanol, Cyclohexanol	[177]
	2020	TD	L: 30 μm	SnO_2_	100 °C	H_2_S	10 ppm	CO, Acetone, Ethanol, Benzene, Toluene	[155]
	2020	TD	L: 10 μm	(TiO_2_ or ZnO) + rGO	RT	Humidity	10%	-	[15]
	2019	TD	W: 100~300 nm, L: 24 μm	rGO	300 °C	HCHO	35 ppb	Ethanol, Acetone, Ammonia, Methanol, Xylene, Toluene	[115]
	2019	TD	W: 80–100 nm, L: 2.5–3 μm	ZnO	RT	NO	10 ppb	NH_3_, CH_4_, H_2_S, NO_2_, H_2_O	[158]
	2019	TD	W: 280-500 nm	OTS-Porous SiNWs	RT	NO_2_	5 ppb	NO, Ethanol, Acetone, Methanol, CH_4_, O_2_, NH_3_	[116]
	2019	TD	W: ~100 nm L: 20 μm	LSiNWs/PPy	RT	NO_2_	50 ppb	Ethanol, Acetone, Methanol, CH_4_, H_2_	[117]
	2019	TD	W: 100 nm	ZnO + Au	300 °C	H_2_S	5 ppm	Ethanol, Acetone, NO_2_	[174]
	2018	TD	D: 100 nm L: 30 μm	PPy	RT	NH_3_	130 ppb	Ethanol, Acetone, Methanol, CH_4_, H_2_	[166]
	2018	TD	D: 200 nm	MoS_2_	RT	NO_2_	1 ppm	-	[164]
	2017	TD	W: 70 nm	OBP	RT	Nonanoic acid	10 ppb	Hexanoic acid	[172]
	2017	TD	L: 35 μm	WO_3_	RT	NO_2_	0.5 ppm	Ethanol, Acetone, CH_4_, NH_3_, H_2_	[159]
	2017	TD	D: 400 nm	TiO_2_	RT	CH_4_	20 ppm	Ethanol, Acetone	[156]
	2017	TD	L: 3.4 μm	MoS_2_	RT	NO	10 ppb	NO_2_, NH_3_, O_2_, H_2_	[163]
	2017	TD	L: 7 μm	GQD	RT	NO_2_	10 ppm	SO_2_, N_2_, O_2_, H_2_	[167]
	2017	TD	No Data	Pd + TeO_2_	200 °C	Benzene	10 ppm	CO, Toluene, NO_2_, H_2_S, Ethanol, Acetone	[175]
	2017	TD	L: 5 μm	MoS_2_	RT	Humidity	11%	-	[162]
	2016	TD	W: 375 nm L: 7.6 μm	ZnO	RT	NO_2_	5 ppm	Methanol, CH_4_, NO_2_	[157]
	2016	TD	D: 400–500 nm, L: 2–2.2 μm	WO_3_	RT	NO_2_	0.25 ppm	Ethanol, Acetone, Methanol, NH_3_, Isopropanol	[160]
	2016	TD	L: 300 nm	Carbon Nanotube	RT	NO_2_	10 ppm	-	[180]
	2014	TD	L: 8 μm	WO_3_	100 °C	NO_2_	2 ppm	Ethanol, Acetone, NH_3_	[181]
	2014	TD	No Data	WO_3_	RT	NO_2_	100 ppb	Ethanol, Methanol, NH_3_, Isopropy alochol	[154]
	2014	TD	D: 50 nm	Graphene	RT	O_2_, H_2_	No Data	-	[168]
FET	2018	TD	W: 100 nm	Amine group	RT	TNT	1 ppb	4-nitrophenol 1,3-dinitrobenzene 2-nitrochlorobenzene	[173]
	2018	TD	W: 150 nm	Pt NPs + POFs	RT	Methanol	No Data	Ethanol, Isopropanol, Acetaldehyde	[178]
	2018	TD	W: 75 nm L: 2 µm	Poly-SiNWs	RT	NH_3_, Ethanol	1–100 ppm	O_2_,H_2_, CH_4_, CO_2_, H_2_O	[182]
	2016	BU	D: 40 ± 8 nm L: 8.5 ± 1.5 μm	Different modification	RT	VOC	Different for every gas	Different VOCs; selectivity depends on learning process and used type of signal	[28]
	2015	BU	D: 40 ± 8 nm L: 8.5 ± 1.5 μm	Propyl, Propenyl, Propynyl	RT	VOC	Different for every gas	Different VOCs; selectivity depends on learning process and used type of signal	[170]
	2015	BU	D: 40 ± 8 nm L: 8.5 ± 1.5 μm	Different modification	RT	Cancer (VOC)	Different for every gas	Different VOCs; selectivity depends on learning process and used type of signal	[171]
	2014	BU	D: 40 ± 8 nm L: 8.5 ± 1.5 μm	Different modification	RT	VOC	Different for every gas	Different VOCs; selectivity depends on learning process and used type of signal	[169]

WT: working temperature, D, L and W stand for diameter, length and width of nanowires.
